# Deep-tissue optical imaging of near cellular-sized features

**DOI:** 10.1038/s41598-019-39502-w

**Published:** 2019-03-07

**Authors:** Xiangnan Dang, Neelkanth M. Bardhan, Jifa Qi, Li Gu, Ngozi A. Eze, Ching-Wei Lin, Swati Kataria, Paula T. Hammond, Angela M. Belcher

**Affiliations:** 1 Department of Materials Science and Engineering, Massachusetts Institute of Technology, Cambridge, MA 02139 USA; 2 The David H. Koch Institute for Integrative Cancer Research, Massachusetts Institute of Technology, Cambridge, MA 02139 USA; 3 Department of Chemical Engineering, Massachusetts Institute of Technology, Cambridge, MA 02139 USA; 4 Department of Biological Engineering, Massachusetts Institute of Technology, Cambridge, MA 02139 USA; 5 Harvard-MIT Health Sciences and Technology, Massachusetts Institute of Technology, Cambridge, MA 02139 USA

## Abstract

Detection of biological features at the cellular level with sufficient sensitivity in complex tissue remains a major challenge. To appreciate this challenge, this would require finding tens to hundreds of cells (a 0.1 mm tumor has ~125 cells), out of ~37 trillion cells in the human body. Near-infrared optical imaging holds promise for high-resolution, deep-tissue imaging, but is limited by autofluorescence and scattering. To date, the maximum reported depth using second-window near-infrared (NIR-II: 1000–1700 nm) fluorophores is 3.2 cm through tissue. Here, we design an NIR-II imaging system, “Detection of Optically Luminescent Probes using Hyperspectral and diffuse Imaging in Near-infrared” (DOLPHIN), that resolves these challenges. DOLPHIN achieves the following: (i) resolution of probes through up to 8 cm of tissue phantom; (ii) identification of spectral and scattering signatures of tissues without *a* *priori* knowledge of background or autofluorescence; and (iii) 3D reconstruction of live whole animals. Notably, we demonstrate noninvasive real-time tracking of a 0.1 mm-sized fluorophore through the gastrointestinal tract of a living mouse, which is beyond the detection limit of current imaging modalities.

## Introduction

Biomedical imaging is an integral part of clinical decision-making during screening, diagnosis, staging, therapy planning and guidance, treatment and real-time monitoring of patient response^1,2^. It remains a major clinical challenge to detect biological features of cellular size with sufficient sensitivity in a complex environment at deep penetration. For instance, identifying small tumor masses before the angiogenic switch growth phase is below the threshold of detection of current imaging technologies^3,4^. There are currently three major limitations to being able to perform whole body imaging at a near-cellular level to allow for inexpensive, real-time imaging at depth: (1) the relative lack of specific, biocompatible probes with excitation and emission in the second near-infrared optical window; (2) the cost of instrumentation with optimal performance in the second optical window; and (3) light attenuation in tissue and blood that is still substantial despite being lessened (by a factor of ~2–3×) relative to that in the visible range^5,6^.

Established clinical imaging modalities do exist, yet these fail to address the aforementioned challenges. These imaging modalities are classified according to the image contrast mechanism as follows: X-ray computed tomography (CT), positron emission tomography (PET), single photon emission computed tomography (SPECT), magnetic resonance imaging (MRI), ultrasound (US), intravital microscopy, fluorescence molecular tomography (FMT), fluorescence lifetime imaging, or some combination of these^2,4^. MRI, while offering good resolution and depth of penetration in tissue, requires large, expensive hardware^7^ and requires long acquisition times (~minutes - hours). Increased exposure to radiation from the widespread clinical prevalence of CT scans^8^ has caused growing concern about the occurrence of radiation-induced cancer^9^. Although ultrasound is a fairly cost-effective technique with a good resolution (~0.2 mm at 7 MHz) close to the point of contact of the probe, it is hampered by poor spatial resolution (~1–2 mm) at increased penetration depth^7^ in tissue, further limited in scope by the poor contrast of ultrasound in soft tissue, making it harder to distinguish between tumor and healthy tissue. Fluorescence-based techniques, such as FMT or lifetime imaging, while offering a wealth of molecular and structural information, are restricted by poor spatial resolution (~few mm) and limited depth of penetration (~few cm) in tissue^10^. Microscopy techniques based on fluorescence (eg., confocal or multi-photon imaging) enable visualization of vascular-level^11–13^ or cellular-level^14,15^ mechanisms; however, they are not suited for either rapid diagnostics at the macroscopic scale or for deep-tissue penetration. Despite significant advances in both imaging instrumentation and algorithms for image processing, most of the aforementioned imaging techniques suffer from a trade-off among sensitivity, resolution, and penetration depth in the three spatial dimensions (3D)^7^, which preclude their applicability in detecting small numbers of cells, for instance, at the very early stages of disease. The most promising technique for high-resolution deep-tissue whole body imaging using relatively safe molecular probes and excitation sources, at a reasonably low cost, appears to be optical imaging.

There has been tremendous interest in the exploration of optical imaging *in vivo*^5^. Fluorescence imaging is of interest due to its high resolution, high sensitivity, and low cost^16^. Visible dye technologies and first-window near-infrared fluorophores (NIR-I: 700–900 nm emission wavelength) have been tested in different preclinical^17,18^ and clinical settings^19^ for the detection of various cancers^20–26^. The advantages of imaging tumors in the NIR domain are: (1) use of non-radioactive molecular probes, and (2) reasonably low cost. However, NIR-I probes suffer from limited tissue penetration and low resolution, which ultimately restricts their efficacy in clinical applications. A study using NIR-I imaging reported a maximum penetration depth through tissue of ~3.2 cm^27^. Another study comparing the imaging performance of quantum dots found a substantial increase in tissue penetration depth (13–1 × 10^6^-fold) for imaging with quantum dots that emit in the NIR-II regime, compared to those that emit in the NIR-I regime^28^. Accordingly, the recent development of various NIR-II fluorescence probes^11–13,29–36^ and custom-built imagers^29,30^ based on InGaAs detectors is promising; however, long-term biocompatibility studies must be done on these probes for clinical translation.

Commercially available whole-animal imagers, such as the Xenogen IVIS Spectrum by Caliper Life Sciences, are optimized for imaging in the visible spectrum and to a certain extent in the NIR-I regime due to their silicon CCD detectors, which have a sharp fall-off in responsivity, and thus usefulness, beyond the NIR-I regime^5^. The lack of commercially available NIR-II whole-animal imagers has necessitated building custom imaging systems using expensive liquid nitrogen-cooled InGaAs focal plane array (FPA) detectors^29,30^, which have a peak quantum efficiency of ~85–90% in the NIR-II regime, despite their intrinsically worse signal-to-noise ratio (SNR) compared to Si detectors (~100 × lower). To compensate for the lower SNR of InGaAs cameras, a more sophisticated data processing algorithm is necessary, and spectral analysis can also be done. For example, *without additional spectral analysis*, when various optical features are detected simultaneously by the camera (Fig. S1a,c in the Supplementary Information), the signals detected from various photo-physical origins are indistinguishable, which in some cases may decrease the SNR and lead to false positives of detection.

Although diffuse light scattering by biological tissues is mitigated in the NIR-II region compared to the visible or NIR-I regions, this scattering would further broaden or perturb the fluorescent signal (Fig. S1c,d), thus imposing a trade-off between the depth of detection and resolution^37,38^ that would impede high-resolution, deep-tissue imaging. For example, a modulated imaging approach has been reported^39^ in the literature, which uses grayscale spatial patterns projected onto the tissue of interest, and fits a modulation transfer function to estimate the average optical properties at each pixel, to obtain estimates of the absorption and reduced scattering coefficients. While this approach has the benefit of a stationary imaging configuration (without the need to raster scan the subject), the sampling depth is limited to ~2–3.3 mm (based on the spatial frequencies used for modulation), and it would be very difficult to apply this method for deep-tissue noninvasive detection of sources of image contrast (such as a fluorescently-labeled tumor, or bacterial infection). To address these challenges, novel imaging methodologies and data processing algorithms for NIR-II fluorescence imaging are highly desirable.

Here, we design an NIR-II optical imaging system that resolves the above challenges of autofluorescence and scattering by performing spectral analysis without requiring *a priori* knowledge of the photo-physical origins of the signal, thus boosting the SNR. This system, named “Detection of Optically Luminescent Probes using Hyperspectral and diffuse Imaging in Near-infrared” (DOLPHIN) achieves the following: (i) deep-tissue detection of 1 mm-sized NIR-II fluorescent probes through up to 8 cm of a breast-mimic optical phantom and 6 cm of tissue; (ii) detection of 100 *μ*m-sized probes through live whole animals for anatomical co-registration with 3D reconstruction; and (iii) noninvasive tracking of a near cellular-sized fluorescent probe through the gastrointestinal tract of a living mouse, which is beyond the detection limit of current imaging modalities. Our initial imaging results with small animals show great promise towards the ability to detect very small tumors down to a cluster of tumor cells, approximately 100 *μ*m in size, which is advantageous for applications such as early detection before the angiogenic switch phase^4^ in cancer formation.

## Results

### Design concept and instrumentation setup

Our DOLPHIN imaging system aims to three-dimensionally reconstruct the fluorescently-labeled tumors located deep underneath the tissue. We achieve this by both exciting the fluorescent probe and detecting the probe emission in the NIR-II region, which is optimally suited for deep *in vivo* optical imaging applications due to the combination of low tissue absorption, low tissue scattering, and low autofluorescence in this wavelength range. We then apply a deconvolution algorithm to the acquired spectral information and diffuse profile of the transmitted light. This algorithm compensates for the autofluorescence and scattering contributions, which precludes the need for *a priori* knowledge of the optical properties of the tissue of interest. This allows us to distinguish different NIR-II fluorescent probes from the spectral information, and, therefore, find the best spectral band for deep-tissue detection. Post-processing on the diffuse profile for the selected spectral band allows for the determination of the probe location.

**Figure 1:**
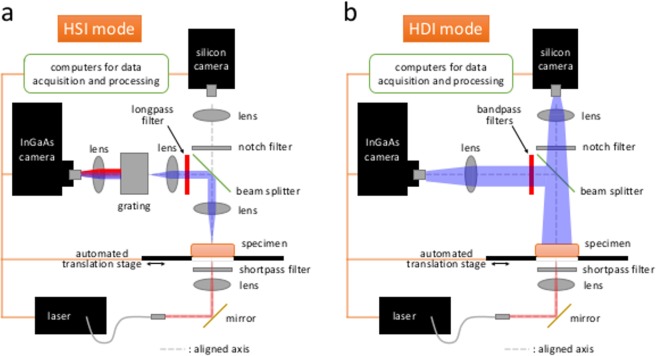
Optical setup of DOLPHIN. **(a)** Hyperspectral Imaging (HSI), and **(b)** Hyperdiffuse Imaging (HDI) modes.

The spectral information and the diffuse profile of the transmitted photons are acquired through two configurations implemented in the DOLPHIN setup: HyperSpectral Imaging (HSI) and HyperDiffuse Imaging (HDI). Both configurations are trans-illuminated, where the specimen is excited with a laser source from the bottom, and the emission signals are acquired from the top. The specimen (which can be an optical tissue phantom, animal tissue, or a whole animal such as a mouse or a rat) is placed on a quartz platform that is attached to an *X*-*Y* translation stage. A 980 nm laser delivers the excitation light from the bottom with a spot size of ~3.5 mm (50 mW of optical power). The photons emitted from the top of the specimen, including residual excitation and fluorescence from the probe, are transmitted by the lens system in the optical path, and detected by a liquid nitrogen-cooled InGaAs camera. Figure 1a shows a schematic of the setup for the HSI configuration, which collects photons from a single point on the specimen surface. These photons are then delivered to a grating system, followed by the InGaAs detector. The combination of the grating system and the InGaAs detector serves the function of a spectrometer, which gives us a full spectrum that ranges from 800–1700 nm. The whole hyperspectral cube dataset (2D image with full spectrum at each point) is accomplished through a 2D raster scan of the physical space, using the translation stage. The excitation and detection optical paths are aligned for better signal detection and ease of analyses. Figure 1b shows a schematic of the setup for the HDI configuration, which collects the 2D diffuse profile of the signal on the surface of the specimen. Compared to the HSI configuration, the grating system is removed so that the 2D diffuse profile can be acquired. An iris is installed in the beam path to minimize stray light from the specimen. Several bandpass filters are chosen based on the results from the HSI analysis. A full 2D raster scan is also performed in HDI mode, with the pixel locations the same as in HSI mode. Meanwhile, a beam splitter is used to split 50% of the transmitted light to a silicon detector for bright-field images. The detailed specifications of the optical components are described in pages S3–S4 of the Supplementary Information.

### Data acquisition and analysis procedure

**Figure 2:**
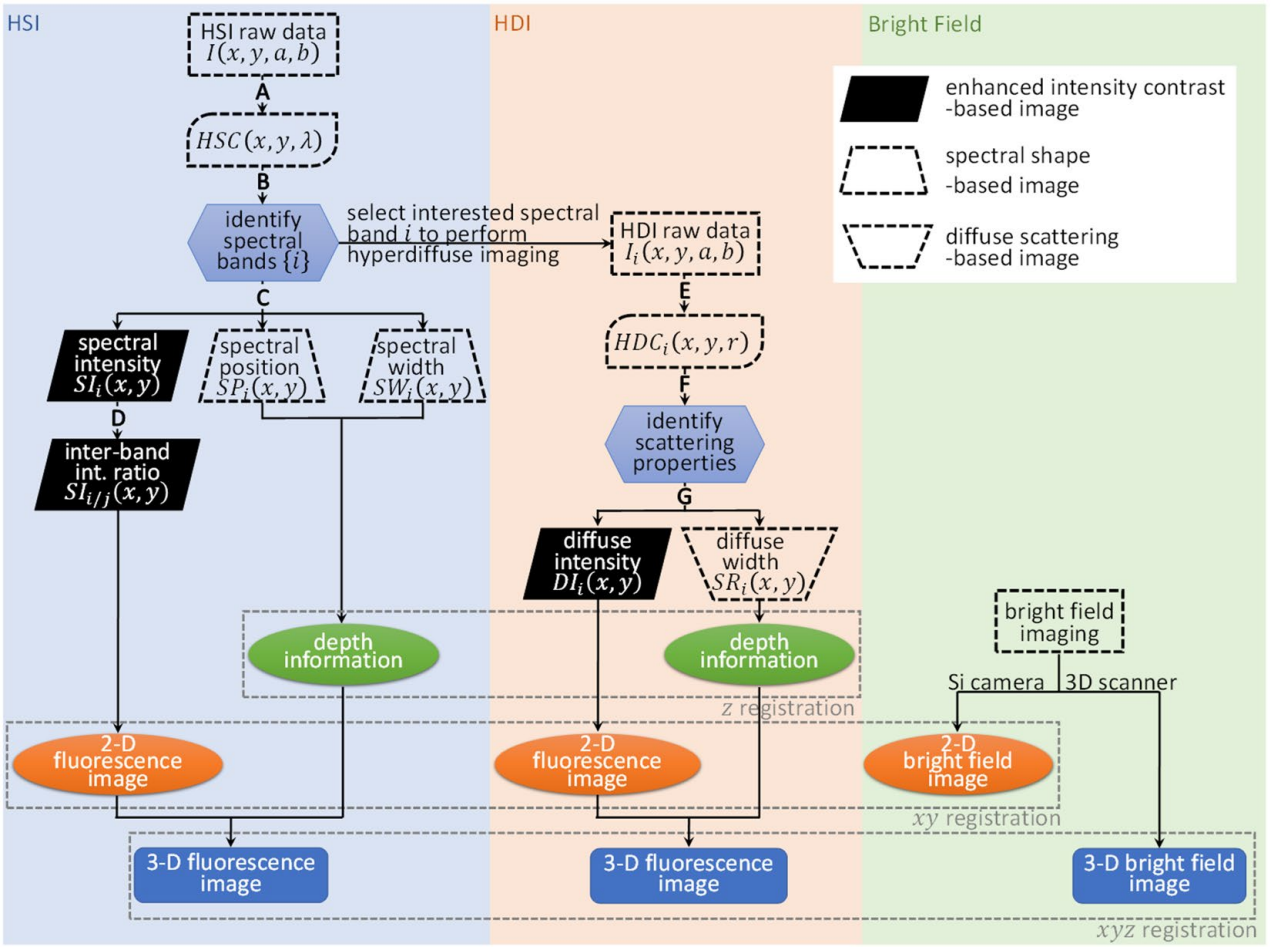
Flowchart of bioimaging with DOLPHIN. This flowchart describes the workflow of data processing for DOLPHIN. Please see the Supplementary Methods for a detailed step-by-step description. **A:** Process raw data to HyperSpectral Cube (HSC). **B:** Band-wise analysis using Principal Component Analysis (PCA). **C:** Intra-band pixel-wise analysis. **D:** Inter-band pixel-wise analysis. **E:** Process raw data to HyperDiffuse Cube (HDC). **F:** Band-wise analysis using PCA. **G:** Pixel-wise diffuse property analysis.

Figure 2 shows the flowchart of the DOLPHIN data processing procedure. The DOLPHIN system acquires 2D camera images at 2D grid points on the specimen. Therefore, the acquired dataset consists of four dimensions. Here, we define the first two dimensions (*x*, *y*) as grid points in real-space on the scanned specimen, and the last two dimensions (*a*, *b*) as pixel locations on the 320 × 256 sensor of the InGaAs detector.

Data processing in HSI mode: The camera images captured using the HSI configuration use only one dimension for spectral dispersion (the long axis), with the range 900–1700 nm covered in 320 pixels, or 2.5 nm/pixel, and the other axis is used to collect the defocused photons with the same photon energy that spreads into the *b* dimension. The first step of data processing reduces the raw data from four to three dimensions by summing along the *b* dimension. We call this new dataset the HyperSpectral Cube, HSC (*x*, *y*, *λ*). Then, principal component analysis (PCA) is performed to deconvolve different emitter sources which emit at different wavelengths. The identified spectral bands are then analyzed through intra-band analyses, termed Spectral Intensity (SI), Spectral Position (SP) and Spectral Width (SW), as shown in the left part of the flowchart in Fig. 2. The mathematical formulation of these methods of analyses is described in detail on pages S4–S6 of the Supplementary Information. Combined information from these three physical parameters (SI, SP and SW) helps us to increase the maximum depth of detection. To help visualization, derived parameters involving inter-band analyses are introduced, termed SI_*i*/*j*_ (page S5 of the Supplementary Information), resulting in enhanced image contrast. The 3D fluorescence image can then be reconstructed based on these processed parameters.

Data processing in HDI mode: The HDI images are acquired using bandpass filters tuned to spectral bands of interest identified from the previous HSI analyses (for example, in our scenario, 4 spectral bands of interest, *viz*. *α*, *β*, *γ*, and *δ* were identified, as discussed in the following section). This is represented in the middle part of the flowchart in Fig. 2. The camera images captured using the HDI configuration show the diffuse profile of the transmitted photons. We first fit the diffuse profiles into a symmetric Gaussian distribution, and reduce the dataset into a HyperDiffuse Cube, HDC (*x*, *y*, *r*). Each HDC_*i*_ (*x*_*i*_, *y*_*i*_, *r*) represents the averaged intensity along radius *r* at a point (*x*_*i*_, *y*_*i*_). Then, PCA is performed to deconvolve different scattering coefficients of the tissue. For a homogeneous tissue phantom (or real tissue with isotropic optical properties, as is assumed for the purposes of this work), there should be only one main principal component. Diffuse Intensity, DI (*x*, *y*) and Scattering Radius, SR (*x*, *y*) are defined from the main (first) principal component. Analogous to the SI in the analysis of the HSI mode, the DI is obtained by summing the intensity of the fitted Gaussian profile, while SR is obtained as the half-width at half-maximum of the Gaussian profile. Combining the information from DI and SR enables us to reconstruct a 2D image, similar to the method applied in the HSI analysis.

Finally, DOLPHIN enables us to reconstruct the 3D fluorescence image using a single 2D detector at a fixed location (unlike conventional fan-beam computed tomography systems, which typically rely on a 360 array of sources and detectors to implement a back-projection algorithm). We achieve this by combining the 2D fluorescence contrast information (obtained from either SI or DI, coupled with bright-field images from the silicon CCD camera), and depth profiles (obtained from either SP and SW in HSI mode, or SR in HDI mode; see Fig. S9 in the Supplementary Information) of the fluorescence signals, as shown in the bottom part of the flowchart in Fig. 2. To calculate the depth profile of the fluorescence signal from SR, we assume the specimen is a homogeneous optical medium. The surface topography of the specimen is obtained using a 3D scanner, which generates a point cloud. The probe location and object profile can then be co-registered with fiduciary markers to visualize the probe location relative to the specimen. We did not perform additional adjustment or motion correction for heartbeat and breathing during live animal imaging. The mathematical definitions of the physical parameters are described on pages S4–S6 in the Supplementary Information.

### Data visualization and spectral band analysis

**Figure 3:**
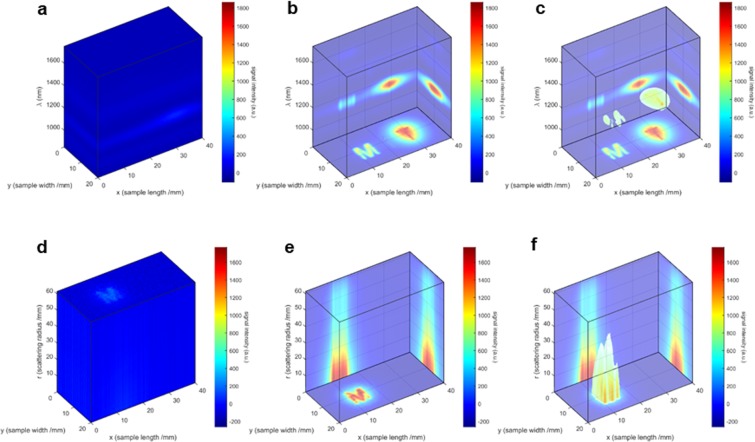
Data visualization using DOLPHIN. Representative 3D visualization of HSC data **(a–c)**, shown here for the *β*-band **(b,c)** as an example, and HDC data **(d–f)**, shown here for the *δ*-band **(e,f)** as an example, by cross-section slicing **(a–f)**. **(a)** *I*(*x*, *y*) at all *λ* are plotted in 3D space by stacking along the wavelength dimension, which emphasizes several spectral bands; **(b)** the summation along each dimension is projected on the back plane respectively; **(c)** combining information from **(a,b)**, a transparency threshold of roughly 0.5 (this threshold has to be arbitrarily determined for each set of HSC, to achieve the best visual effect) is applied to visualize the features in 3D space; **(d)** *I*(*x*, *y*) at all *r* are plotted in 3D space by stacking along the dimension of the scattering distance; **(e)** the summation along each dimension is projected on the back plane respectively; **(f)** combining information from **(d,e)**, a transparency threshold of roughly 0.5 (this threshold has to be arbitrarily determined for each set of HDC, to achieve the best visual effect) is applied to visualize the features in 3D space. Refer to Fig. 2 for the definitions of HSC and HDC. See Fig. 4 for the definitions of the *β*- and *δ*-bands.

**Figure 4:**
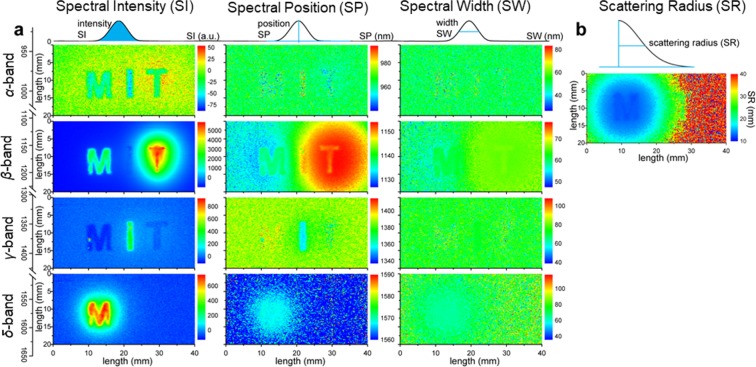
Spectral and Scattering analyses of HSC and HDC for tissue penetration. **(a)** Band-wise spectral analyses: Spectral Intensity (SI), Spectral Position (SP) and Spectral Width (SW) (plotted column-wise, left to right), of the four spectral bands (*α*, *β*, *γ*, and *δ*, plotted row-wise, top to bottom) identified through PCA of HSC. The schematics explaining the concepts of SI, SP and SW are shown on the top, while the illustration of the spectral bands and the corresponding wavelength ranges are shown on the left of each sub-figure. **(b)** Scattering Radius (SR) of HDC based on the scattering profiles. The schematic explaining SR is shown on the top. The spectral and scattering analyses are performed for each (*x*, *y*) location. See pages S5–S6 of the Supplementary Information for the mathematical definitions of SI, SP, SW and SR.

Figure 3 shows an example of the 3D data visualization techniques used to observe the outputs of the HSI and HDI imaging techniques. For ease of comparison, only one representative band is shown for each case of HSC and HDC. Figure 3a–c represent HSC analysis for the *β*-band, while Fig. 3d–f represent HDC analysis for the *δ*-band (see the paragraph with the description of Fig. 4 below, for the definitions of all 4 bands). To visualize the major spatial, spectral, and scattering features of the HSC and HDC, volume-rendering techniques of cross-section slicing and maximum intensity projection are employed. In Fig. 3b, the image shown on the *XY* plane is the summation ${\sum }_{\lambda =900\,{\rm{nm}}}^{1700\,{\rm{nm}}}\,I(x,y,\lambda )$ along the *Z*-axis (*ie*. along the *λ*-axis), and correspondingly ${\sum }_{x=0\,{\rm{mm}}}^{40\,{\rm{mm}}}\,I(x,y,\lambda )$ and ${\sum }_{y\mathrm{=0}\,{\rm{mm}}}^{20\,{\rm{mm}}}\,I(x,y,\lambda )$ for the images shown on to the *YZ* and *XZ* planes, respectively. In Fig. 3e, the image shown on the *XY* plane is the summation ${\sum }_{r=0\,{\rm{mm}}}^{60\,{\rm{mm}}}\,I(x,y,r)$ along the *Z*-axis (*ie*. along the *r*-axis), and correspondingly ${\sum }_{x=0\,{\rm{mm}}}^{40\,{\rm{mm}}}\,I(x,y,r)$ and ${\sum }_{y=0\,{\rm{mm}}}^{20\,{\rm{mm}}}\,I(x,y,r)$ for the images shown on to the *YZ* and *XZ* planes, respectively. It should be noted here that a transparency threshold of 0.5 has been applied to Fig. 3c,f (with the threshold being determined arbitrarily, to achieve the best visual effect).

The bottom projection emphasizes the spatial features by combining all information about frequency or scattering domain (along the *Z*-axis or *λ*-axis), and simulates the result obtained from conventional fluorescence imaging, which collects optical signals over the range of the spectrum controlled by the optical filters and the scattering distance controlled by the spatial filters (ie., apertures). Meanwhile, the side projections highlight the spectral or scattering information. As a result, the spectral and scattering information collected by the DOLPHIN system can be independently analyzed in HSI or HDI mode respectively, enabling us to perform a 3D reconstruction of the size, location, and depth of the fluorophore.

Figure 4 shows an example of 2D visualization of the complete analyses of the HSC data (through SI, SP, and SW plots, Fig. 4a), and the HDC data (through the SR plot, Fig. 4b). Shown above each column are schematics of the concept of Spectral Intensity (SI), Spectral Position (SP), Spectral Width (SW) and the Scattering Radius (SR). These analyses were done for three probes, forming the 3 letters of “MIT”: “M” being NaYF _4_:Yb:Er (Er-NP), “I” being NaYF _4_:Yb:Pr (Pr-NP) and “T” being NaYF _4_:Yb:Ho (Ho-NP), which were placed directly underneath the tissue at a fixed depth (2 cm in breast-mimic optical phantom). See Fig. S14 in the Supplementary Information for the spectra of these three probes. For the spectral analyses in Fig. 4a, four bands of interest were identified from PCA of the HSC data: *α*, *β*, *γ*, and *δ* bands. These 4 bands are attributed to various light-probe-tissue interactions, with the principal components (PCs) identified as follows: (1) *α*-band (~980 nm, PC 5): represents contrast by the excitation light; (2) *β*-band (~1100 nm, PC 1, 2 and 4): arises from small Stokes’ shift autofluorescence (<2000 cm^−1^) and probe emission of the Er-NP and Ho-NP; (3) *γ*-band (~1350 nm, PC 3, 5): originates from large Stokes’ shift autofluorescence (>2500 cm^−1^) and probe emission of Pr-NP; and (4) *δ*-band (1600 nm, PC 2): represents the probe emission of Er-NP. Our visualization techniques reveal DOLPHIN’s ability to multiplex imaging various sources simultaneously, followed by choosing a specific probe at the deconvoluted spectral band of interest for further investigation using a suitable image processing algorithm. For example, in Fig. 4a, the SI analysis provides the best visual contrast for the *β*- and *δ*-bands, while the SP analysis works best for the *γ*-band. It is worth noting here that Fig. 4 is an alternative form of visualization of the 3D representation in Fig. 3, with the Fig. 3a–c corresponding to the SI analysis of the *β*-band, and Fig. 3d–f corresponding to the SI analysis of the *δ*-band. This analysis shows the true benefit of the DOLPHIN method compared to existing imaging systems, without having *a priori* knowledge of the source of the signals detected in deep tissue.

### Sensitivity and depth of detection of DOLPHIN

**Figure 5:**
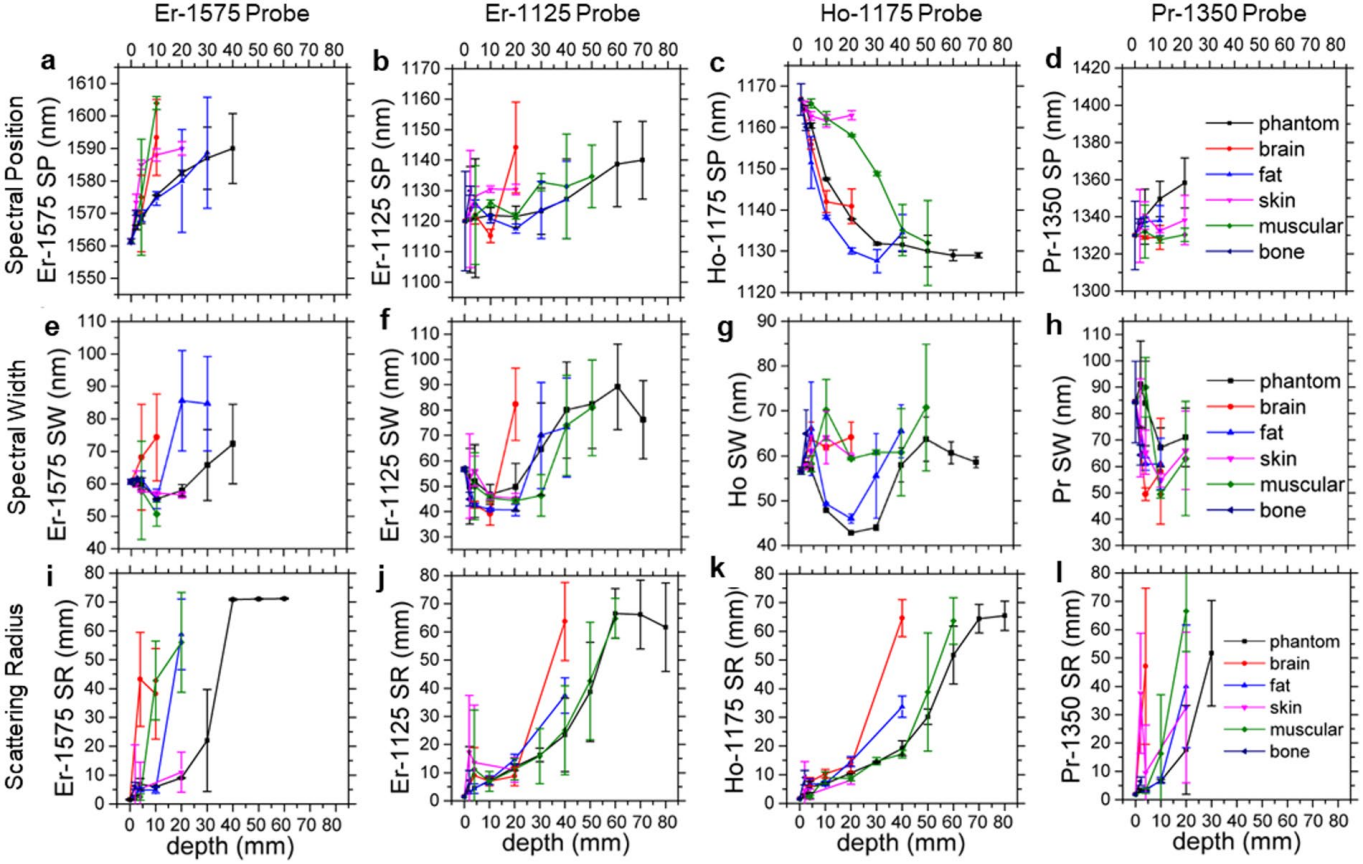
Effects of thickness and type of tissue on the spectral and scattering properties identified by DOLPHIN. Six types of tissues are studied, including breast-mimic optical phantom (black), brain (red), fat (blue), skin (pink), muscle (green) and bone (purple). Depending on the type of tissue, the tissue thickness studied varies from 2 mm to 80 mm. The spectral position (SP) **(a–d)**, spectral width (SW) **(e–h)** and scattering radius (SR) **(i–l)** of four probes with distinct NIR emissions, Er-1575 **(a,e,i)**, Er-1125 **(b,f,j)**, Ho-1175 **(c,g,k)** and Pr-1350 **(d,h,l)** are presented. Data shown are mean ± s.d. for *n* ≥ 10 samples (pixels used for calculation, see Supplementary Information for the sample sizes of this figure) at each depth, tissue type and probe condition.

Having developed the analysis techniques of SI, SP, SW and SR, we subsequently applied them to study the sensitivity and the maximum depth of detection possible using the DOLPHIN technique, through various kinds of representative biological tissues. The tissues studied were: breast-mimic optical tissue phantom, brain, fat, skin, muscle, and bone tissues obtained from a cow slaughtered in an abattoir (with the exception of the tissue phantom). Different thicknesses of the various tissues (corresponding to depths of 2, 5, 10, 20, 30, 40, 50, 60, 70 and 80 mm) were achieved by slicing layers of the tissue with a kitchen knife, or by machining the tissue phantom. The HSI and HDI images were obtained by placing a 1 mm-sized cluster NP probe of the corresponding nanoparticle (Er-NP, Ho-NP, or Pr-NP probes) under the various depths of tissues listed above, and the results of the SP, SW and SR analyses are plotted in Fig. 5.

In terms of the Spectral Position, SP: the shift in the *β*-band Ho-1175 nm spectral peak (Fig. 5c) can be explained by the presence of a strong differential absorption at 1175 nm in muscle tissue. In terms of the Spectral Width, SW: we do not observe significant changes as a variation of the depth of tissue penetration, for the *γ*- and *δ*-band (Fig. 5e,h respectively), as there is no variation (local maximum) in the attenuation coefficient around these wavelengths. However, we see a general upward trend in the *β*-band (Er-1125 nm), which can be correlated to the depth of tissue up to 6 cm in breast-mimic phantom, and up to 5 cm in muscle tissue (Fig. 5f). In terms of the Scattering Radius, SR: we observe a general increasing trend with the depth of tissue penetration, for all kinds of tissues tested, for all bands (Fig. 5i–l). However, the maximum depths of detection vary with the type of tissue and the band used. We report a maximum depth of detection through 8 cm of breast-mimic tissue phantom, and through 6 cm in muscle tissue (Fig. 5j,k). While most types of tissue examined exhibit similar diffuse scattering properties at comparable penetration depths, muscle and brain tissues scatter more strongly than other types, underscoring the challenges involved in performing high-resolution, deep-tissue imaging in these tissues. For instance, the maximum depth of detection reported through brain tissue is ~2 mm^13^; however, DOLPHIN allows for probe detection at a penetration depth of ~40 mm in brain tissue, in HDI mode (Fig. 5k). We note here, that for the cases of Fig. 5i–k, the data plotted for the SR through the breast-mimic tissue phantom (black curves) shows saturation. The immediate result of the saturation is that for depths greater than the saturation depth (40 mm using the Er-1575 probe, 60 mm using the Er-1125 probe, or 70 mm using the Ho-1175 probe, respectively), the SR analysis cannot be used to reliably predict the depth of the fluorescent signal. However, we note that this saturation occurs in a very small fraction of all the data points observed, and is only limited to the SR mode of analysis. A physical explanation as to the origins of the signal saturation can be attributed to the finite physical size of the tissue phantoms used in this study (the largest dimension phantom used in this study was 9 cm × 9 cm × 1 or 2 cm thickness, with multiple such units stacked together to achieve a total depth of 80 mm, as described on page S28 of the Supplementary Information). Such a geometry does not present a semi-infinite scattering medium in the *XY* dimension. When photons scatter through greater depths, they require a larger area to calculate the scattering radius; otherwise, edge effects begin to play a significant role in the measurement and the subsequent analysis. Since saturation is observed for the SR data points of the tissue phantom, these SR values should not be used for calculating the depth of the fluorophore. The purpose of Fig. 5 is to show the various possible ways to predict the depth of the sources of various fluorescent signals emitted, based on the spectral (SP, SW) and scattering (SR) features. As with any predictive model, however, there are limitations to the range of depths to what these parameters can be used to predict, and should be used with caution to avoid spurious prediction due to edge effects and other sources of physical interference.

Upon performing the analyses to a variable-depth scenario, for a given tissue type and probe fluorescence, comparison of SI, SP and SW reveals a spectral band that is optimally suited at a given depth. This type of analysis allows DOLPHIN to achieve the maximum depth of detection possible. For example, to image through tissue phantom with the Er-NP cluster probe, analysis of the SI, SP and SW plots reveals the following: for depths between 0 and 20 mm, the *δ*-band dominates, whereas for depths greater than 30 mm, the *β*-band has a stronger signal while the *δ*-band falls off (see Fig. S10 in the Supplementary Information). Compared to the *γ*-band and the *δ*-band, the *β*-band has a small spectral separation from the excitation wavelength, which is more difficult to resolve from the laser source and therefore results in lower SNR^40^. However, another dominating source of background noise, which is autofluorescence, is lower in DOLPHIN because of the trans-illumination configuration, which enables us to achieve deeper depth of detection compared to conventional epi-fluorescence imaging systems. Therefore, DOLPHIN can use the *β*-band to image up to 80 mm through tissue phantom, 60 mm through muscle tissue, and 20 mm through brain tissue with the Er-NP cluster probe (Fig. 5b,f). From our analyses, we suggest that the best possible combination of excitation source and probe emission are: using a 980 nm laser source, and ~1100–1200 nm *β*-band emission (from the Er-NP or Ho-NP) respectively, in order to achieve the maximum possible depth of detection for various kinds of tissues.

**Figure 6:**
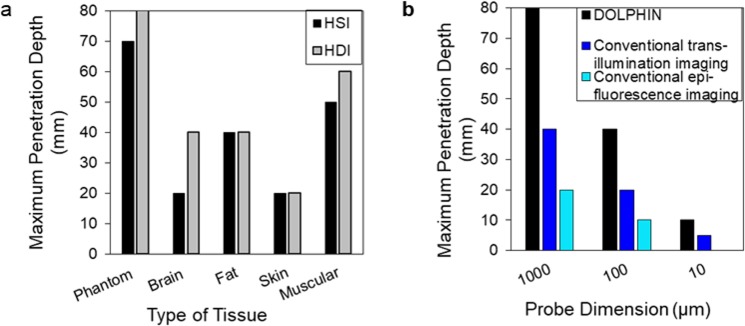
Effect of tissue type and probe size on maximum depth of detection, and comparison of DOLPHIN with conventional imaging systems. **(a)** The maximum depth of detection through breast-mimic optical phantom achieved by HSI (black) and HDI (gray) for various types of tissues studied (breast-mimic optical phantom, brain, fat, skin, and muscle). **(b)** A comparison of the maximum depth of detection achieved through breast-mimic optical phantom, using DOLPHIN (black), conventional NIR-II imaging performed in trans-illumination (blue) or in epi-fluorescence (cyan) modes. For **(a)** or **(b)**, the maximum depth of detection is achieved when at least one of the “M”, “I”, or “T” letters can be detected and identified. For **(b)**, each probe dimension represents an estimate of the actual size of the probe, and the actual dimension is at most 2× larger than the indicated size.

In Fig. 6, we summarize the results of the DOLPHIN imaging technique, through a study of the effects of the type of tissue (Fig. 6a) and the probe cluster size (Fig. 6b) on the maximum depth of detection. In Fig. 6a, we do a comparison of the maximum depth through various kinds of tissues, for HSI and HDI imaging modes, through breast-mimic tissue phantom, brain, fat, skin and muscle. Notably, for all major types of tissue examined, except for skin, the maximum depths of detection are greater than 4 cm, in particular: 8 cm and 6 cm for breast-mimic phantom and muscle tissue, respectively, from HDI, and 7 cm and 5 cm for breast-mimic phantom and muscle tissue, respectively, from HSI (Fig. 6a). The greater depths of detection observed with HDI mode are made possible because of the ability to exclude much of the diffuse scattering of the probe emission from the resulting contrast images. It is worth highlighting here that the ability to detect light penetrating through ~8 cm of phantom, or through 6 cm of muscle tissue is significantly more improved compared to the previously reported maximum depth of detection of ~3.2 cm in pork tissue^27^. Nonetheless, we believe that the penetration depth with DOLPHIN could be further improved by using optimized fluorescent probes with higher quantum yields, better imaging optics, and more advanced processing algorithms. In summary, we have demonstrated a major advantage of DOLPHIN, which is the capability to resolve the effects of scattering and tissue autofluorescence to maximize the depth of probe detection. We consider DOLPHIN to be a promising platform for the detection of near cellular-sized features through deep biological tissues, that could be suitably applied to track fluorescent probes or fluorescently-labeled cells in whole animals.

Further, we estimate the minimum number of fluorescently-labeled cells required for reliable detection, using our DOLPHIN imaging system (pages S14–S15 of the Supplementary Information). In a realistic model of tumor tissue, with cells fluorescently-labeled through uptake of ~100 Er-NPs per cell, with a cell size of ~20 *μ*m, we calculate that the minimum tissue sizes required for detection are: 27 cells, 979 cells (~0.2 mm-sized tumor) and 5919 cells (~0.36 mm-sized tumor), for detection through 2 cm, 6 cm, or 8 cm of human breast tissue respectively. Therefore, we believe that DOLPHIN opens up the possibility of detecting signal from few-cell clusters, at depths of up to 2 cm, and from sub-millimeter sized tumors, through up to 6–8 cm of breast tissue.

### *In vivo* Tracking of a Fluorescence Cluster *via* 3D reconstruction

**Figure 7:**
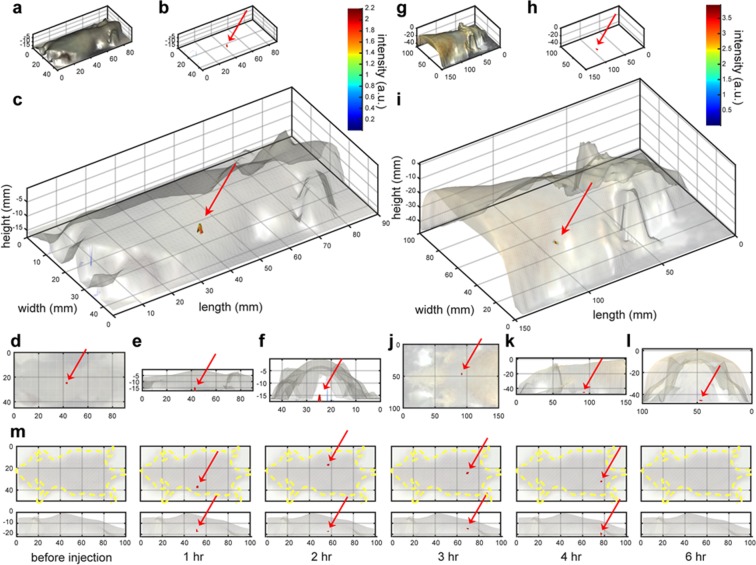
3D Reconstruction of *in vivo* tracking of a fluorescence cluster probe. **(a–f)** 3D reconstruction of the fluorescence signal of a 100 *μ*m-sized Er-NP cluster probe detected through a whole mouse, through approximately 2 cm thickness of body; **(g–l)** 3D reconstruction of the fluorescence signal of a 1 mm-sized Er-NP cluster probe detected through a rat, through approximately 4 cm thickness of body. **(a,g)** Surface profiles of the animals measured by a 3D scanner, *ie*. 3D bright-field images; **(b,h)** the reconstructed height profiles of the fluorescence signals measured from HDI, *ie*. 3D fluorescence images; **(c,i)** overlays of the 3D bright-field and fluorescence images, with a transparency of 0.1–0.2 applied to the bright-field surface images to better visualize the reconstructed fluorescence images in 3D; (**d–f,j–l**) are top views along the *Z*-axis **(d,j)**, side views along the *Y*-axis **(e,k)** and side views along the *X*-axis **(f,l)** of the 3D overlay images in **(c,f)** respectively. **(m)** Tracking of a 100 *μ*m-sized Er-NP cluster probe in the gastrointestinal tract of a whole mouse after oral administration. The top row shows the dorsal positions (yellow dotted lines are drawn to indicate the outline of the mouse), while the bottom row shows the lateral positions. The fluorescence of the probe cluster is observed in the stomach (1 hr.), small intestine (2–3 hr.), and large intestine (3–4 hr.). No fluorescence signal is detected before oral dosing, or at 6 hr. after administration, at which point it has been excreted from the body. The red arrows in (**b–f**,**h–m**) are drawn to help identify the location of the fluorescent probe.

Finally, we demonstrate tracking of a cluster probe inside a living mouse. We detected a 100 *μ*m-sized Er-NP cluster probe through the whole body of a mouse (~2 cm thick, Fig. 7a–f, Movie S3), and a 1 mm-sized Er-NP cluster probe through the whole body of a rat (~4 cm thick, Fig. 7g–l). A 100 *μ*m-sized Er-NP cluster probe, which is stable against acidic or basic conditions, is tracked inside the gastrointestinal tract of a living mouse after oral administration to the esophagus, and the fluorescent probe is observed in the stomach (1 hr.), small intestine (2–3 hr.), and large intestine (3–4 hr.) at various time points (Fig. 7m). As a comparison, we used our home-built NIR-II epi-fluorescence setup to image the small NIR-II fluorescent probes in the gastrointestinal tract of a mouse, but no observable signal is found (data not shown). This demonstrates the sensitivity of our DOLPHIN technique, which surpasses that of a conventional epi-fluorescence configuration. Tracking of cellular-sized features is important for clinically translational research such as monitoring immune cell trafficking in response to inflammation or cancer immunotherapy. The ability to track populations of immune cells would provide critical insights into the factors affecting the success of treatment, and thus improve our ability to intervene and design better treatment regimens.

## Discussion

We first compare DOLPHIN to current state-of-the-art imaging and data processing technologies. To the best of our knowledge, DOLPHIN is the first demonstration of utilizing both HSI and HDI modes in a trans-illumination configuration to investigate NIR-II fluorescent signals. In contrast, previous HSI^41,42^ and HDI^43^ technologies worked mainly in the visible and NIR-I wavelengths, utilized either epi-illumination or reflectance configurations that result in shallower depths of detection, and also relied on mapping to reference spectra to identify features of interest. Some recent studies^44,45^ have observed the spectral *β*-band (~1100–1200 nm) for NIR-II trans-illumination imaging up to 20 mm in depth; the authors used a limited quantity of probe which may explain the shallow depth of detection. In one notable approach, a recent study^42^ has reported the instrumentation design for a wide field-of-view, time-resolved hyperspectral imaging system with high sensitivity, which was used to quantify the fluorescence intensity and mean lifetime of Förster resonance energy transfer (FRET), both *in vitro* and *in vivo*. However, DOLPHIN differs from this approach in three important ways: (a) DOLPHIN utilizes NIR-II HSI and HDI modes of imaging, in the range of 900–1700 nm, while the previous study was focused on the NIR-I region of 720–800 nm (see the Introduction for a discussion of the preferred choice of NIR-II over NIR-I for biomedical imaging applications); (b) DOLPHIN is based on a trans-illumination configuration, while the previous study used a reflectance geometry for the *in vivo* measurements; and (c) DOLPHIN is focused on the use of SP, SW and SR analyses of the HSI and HDI data to obtain depth information for deep-tissue detection of fluorescent signals, while the previous study was focused on the use of time-domain information for detecting and quantifying energy transfer processes. Building on the previous study, another recent study^46^ outlines the use of a deep-learning approach using a convolutional neural network (CNN) called “Net-FLICS” (Network for Fluorescence Lifetime Imaging with Compressive Sensing), which aims to fast reconstruct (in near real-time) the intensity and lifetime images directly from time-resolved data. However, this neural net was trained on a hand-written digits recognition sample, and has yet to be tested on images of real biological tissues. To the best of our knowledge, observation of the spectral *β*-band to detect up to 80 mm in tissue phantom, 60 mm in muscle tissue, and up to 40 mm in fat or brain tissues (HDI mode data), has not been reported or applied in prior studies.

Other studies have employed the emission of Er-NP in the *δ*-band (~1550 nm), and have used an epi-fluorescence configuration, which precluded their ability to image more deeply than ~32 mm. To illustrate this point, in Fig. 6b we compare the depth of detection of the DOLPHIN technique with conventional trans-illumination and epi-fluorescence imaging modes, for different sizes of the Er-NP cluster probe (comprising 1 mm, 100 *μ*m and 10 *μ*m in diameter). The “conventional trans-illumination” data was obtained by laser illumination on the same side as the fluorescent probes and opposite to the detector, while the “conventional epi-fluorescence” data was obtained by laser illumination on the same side as the detector (see page S25 of the Supplementary Information for details). Compared to these conventional imaging modes, DOLPHIN greatly enhances the maximum depth of detection for all probe sizes (Fig. 6b), and demonstrates the feasibility of detection of 100 *μ*m-sized Er-NP cluster probes through 4 cm, or 10 *μ*m-sized Er-NP cluster probes through 1 cm of breast-mimic phantom.

A trans-illumination configuration, as implemented in DOLPHIN, has the benefits of^47^: (i) collecting more spectral and scattering information through greater interaction of the excitation light with the bulk tissue; (ii) more homogeneous detection sensitivity through a range of depths of location of the fluorophore, than in epi-illumination configurations (which are inherently better suited to detect fluorophores at shallow depths from the surface); and (iii) having minimal tissue autofluorescence interfere with the probe signal, compared to epi-illumination (see page S19 of the Supplementary Information for a detailed discussion). For clinical translation to humans, a trans-illumination configuration would be desirable for certain situations, such as for the diagnosis of breast cancer, or for real-time fluorescence-guided surgery, where the advantages (*eg*., low tissue autofluorescence, high sensitivity) outweigh the disadvantages (more complicated instrumentation setup compared to epi-illumination).

Notably, the ability to detect and track 100 *μ*m-sized cluster probes through a whole animal is valuable for developing technology for clinical translation to detect very early-stage tumors. For example, a single HeLa cell has a size of ~20 *μ*m in diameter^48^. Therefore, DOLPHIN can detect an early-stage 100 *μ*m-sized primary tumor, which corresponds to a volume of ~200 cells. This level of sensitivity has not been shown previously using noninvasive fluorescence imaging methods, or other imaging modalities such as X-ray CT. Clinical CT, for comparison, has a resolution limitation^49^ of 600 × 600 × 600 *μ*m^3^, which is not high enough to resolve microscopic tumors for early detection or diagnosis. Another comparable imaging system, fluorescence molecular tomography (FMT), has a detection limit of ~1 mm, at a depth of ~1.5 cm in tissue (see pages S20–S21 of the Supplementary Information for a detailed discussion on FMT). In this regard, DOLPHIN can potentially significantly enhance the use of fluorescence imaging in the clinic for deep-tissue detection of challenging, near cellular-sized features.

In conclusion, we have designed a next-generation imaging system, “DOLPHIN”, that (a) significantly surpasses the maximum reported depth of detection (~3.2 cm) through biological tissue, using optical imaging, and (b) enables the detection and noninvasive tracking of near cellular-sized features, which, upon clinical translation, would allow for the detection of microscopic tumors and potentially improve patient outcomes. This system combines the advantages of NIR-II fluorescence imaging in a trans-illumination configuration, with the dual-mode spectroscopic analyses of hyperspectral and hyperdiffuse imaging. The versatility of DOLPHIN is demonstrated in the following results, through the capability of: (i) detecting 1 mm-sized particles through up to 8 cm of a breast-mimic phantom, or through a whole live rat, (ii) locating and noninvasively tracking 100 *μ*m near cellular-sized particles through the gastrointestinal tract of a live whole mouse, which is beyond the detection limit of current imaging modalities. Given these advancements, this study opens up exciting new possibilities for clinical translation of NIR-II imaging as a viable platform for theranostic technology; for early diagnostics, as a real-time surgical assistance tool, and for monitoring patient response to therapies.

## Methods

Please refer to the Supplementary Information file, pages S3–S29, for detailed methods.

### Ethics statement regarding the use of experimental animals

All *in vivo* experiments were performed in compliance with the Institutional Animal Care and Use Committee protocols. Animal experiment procedures were pre-approved (Protocol #1215-112-18) by the Division of Comparative Medicine (DCM) and the Committee on Animal Care (CAC), Massachusetts Institute of Technology, and in compliance with the Principles of Laboratory Animal Care of the National Institutes of Health (NIH), United States of America.

## Supplementary information

Supplementary Information

Movie S1

Movie S2

Movie S3

## Data Availability

All MATLAB files associated with the data processing used in this work are available online in the GitHub repository: https://github.com/belcherlab/DOLPHIN. A small test dataset is stored online, in the Dryad Digital Repository (10.5061/dryad.dr7d18t). The full dataset will be made available to interested researchers upon reasonable request. Requestors must provide their own portable storage solution (in the form of a flash drive or portable hard drive) with sufficient capacity ~1 TB, and pay for return shipping.
