# Supplementary material for: Deep-tissue optical imaging of near cellular-sized features
Source: Sci Rep. 2019 Mar 7;9:3873. doi: 10.1038/s41598-019-39502-w (PMC6405836; doi:10.1038/s41598-019-39502-w)
Supplement: Supplementary file 1 — Supplementary Information [file 41598_2019_39502_MOESM1_ESM.pdf]

Supplementary Information

Deep-tissue optical imaging of near cellular-sized features

Xiangnan Dang[1,2,#], Neelkanth M. Bardhan[1,2,4,#], Jifa Qi[1,2], Li Gu[2,3], Ngozi A. Eze[2], Ching-Wei Lin[2], Swati Kataria[2,5], Paula T. Hammond[2,3], and Angela M. Belcher[1,2,4]*

[1]Department of Materials Science and Engineering, Massachusetts Institute of Technology, Cambridge, MA 02139, USA
[2]The David. H. Koch Institute for Integrative Cancer Research, Massachusetts Institute of Technology, Cambridge, MA 02139, USA
[3]Department of Chemical Engineering, Massachusetts Institute of Technology, Cambridge, MA 02139, USA
[4]Department of Biological Engineering, Massachusetts Institute of Technology, Cambridge, MA 02139, USA
[5]Harvard-MIT Health Sciences and Technology, Massachusetts Institute of Technology, Cambridge, MA 02139, USA

# These authors contributed equally

* Correspondence should be addressed to: belcher@mit.edu

Supplementary Materials:

Supplementary materials and methods	S3
Supplementary Figures S1-S15	S30
Supplementary movies S1-S3	S42
Supplementary Code	S42
Supplementary references S1-S34	S43

Supplementary materials and methods:

Instrumentation of DOLPHIN:

In DOLPHIN, a computer-controlled, fibre-coupled laser in NIR-I (such as 808 nm or 980 nm) is collimated and filtered (via multiple short pass filters) before illuminating the target medium. If necessary, the collimated laser beam passes through lenses and mirrors to adjust the angle and size of the laser beam. The operation of the laser is controlled by computer to avoid unnecessary laser illumination of tissues or animals. On a computer-automated *X-Y* translational stage, phantom, tissue, or whole animals are placed on a supporting quartz plate, which minimizes the autofluorescence signal compared to other supports. For hyperspectral imaging (HSI) mode, a pair of convex lenses is used to collect light from the surface of the sample to the entrance slit of a monochromator, which is normally set at 0.5 mm. The monochromator is equipped with a 150 lines/mm NIR diffraction gratings (Newport), to split the collected NIR signal from 900-1,700 nm. At the exit of the monochromator, no slit is used and the split light is coupled in the liquid nitrogen-cooled InGaAs camera (Princeton Instrument, OMA:V 2D, 256 × 320 pixel array used without binning, detection range: 900-1,700 nm, temperature –100 °C) by a pair of achromatic doublets (with anti-reflection coating of 1,050-1,650 nm). For hyperdiffuse imaging (HDI) mode, a short-wave infrared (SWIR) camera lens (SWIN-25, Navitar) is used to collect light from the surface of the sample to the liquid nitrogen-cooled InGaAs camera. For both HSI and HDI modes, filters are placed between the sample and the camera to prevent the direct or scattered laser light from interfering with the specimen signal. Additional bandpass filters are used for HDI mode, to selectively image various spectral band components. In addition, a silicon camera (Hamamatsu, ORCA-Flash4.0 LT) with a camera lens (MVL25M1, Navitar) is used for alignment and capturing white-field images. A beam-split mirror is used to split collected light for both InGaAs and silicon cameras. While we use one computer to control the laser, automated stage, and the operation of the cameras, the captured data and images are directly stored on a second computer, which also handles all the data processing in real-time, as well as post-processing. The excitation source and (spectral) imaging components are fixed, while the tissue sample with the fluorescent probe is placed on an automated *XY* translational stage (assembled from one NRT100 stage and one LTS300 stage, Thorlabs). The 2D spatial scanning operation on the tissue sample improves spatial

resolution and allows us to study and decouple the effect of diffuse scattering from probe fluorescence, thus boosting the signal-to-noise ratio (SNR).

When the collimated laser beam is pre-aligned with light collection elements in the trans-illumination configuration, the target medium being imaged is located in an optical path between the illuminating light source and the detector array. Alternatively, the system could also be configured in reflection configuration, wherein the illuminating laser is located on the same side as the detector array, with the image being collected after interaction of the irradiant light with the target medium and other features of interest.

Flowchart of bioimaging with DOLPHIN:

This section explains the flowchart step-by-step, for mathematical processing and examples. From "A" to "G", the corresponding steps can be found in the flow chart (**Figure S3**):

A: $I(x, y, a, b) \rightarrow I(x, y, \lambda, b) \rightarrow I(x, y, \lambda)$: HSC(x, y, λ)

The directly measured results (raw data) of $I(x, y, a, b)$ at (*e.g.*, 200 × 100) positions (x, y) comprised of 320 × 256 intensity pixels (a, b) were transformed to a hyperspectral cube (HSC) of 320 spectral bands (later processing uses the spectral bands 1 – 260), HSC, and $I(x, y, \lambda)$, where λ is the wavelength.

B: [Coeff(λ), Score(x, y), Explained] = PCA(HSC(x, y, λ))

with the functional domains for the three parameters defined as Coeff(λ, components = 1-6); Score(x, y, components = 1-6), which are the contrast images; and Explained(components = 1-8), which are the contribution factors. The principal component analysis (PCA) is performed by the *pca* function in MATLAB.

The first parameter, Coeff, contains information describing the transformation of principal components (PCs) from spectral bands. Coeff of the first 6 PCs (ordered by the relative contribution from each component to the HSC) are plotted (**Figure S5d**) to help identify the most pronounced spectral bands. We have identified 4 bands based on PCA and the light-probe-tissue interaction as follows: (1) α-band (~980 nm, PC5) represents contrast by excitation light, (2) β-

band (~1100 nm, PC1,2,4) arises from low-wavenumber tissue autofluorescence (<2,000 cm^{-1}) and probe emission of Er-NP and Ho-NP, (3) γ-band (~1,350 nm, PC3,5) originates from high-wavenumber tissue autofluorescence (>2,500 cm^{-1}) and probe emission of Pr-NP, and (4) the δ-band (~1,600 nm, PC2) represents probe emission of Er-NP. The second parameter, Score (**Figure S5e**), contains the linear combination processed image from each PC listed in order of contribution; most information is contained in the first five components and the rest are dominated by noise. The third parameter, Explained, describes the contribution from each PC to the measured results, HSC(x, y, λ), as shown in **Figure S5d, *inset***. Depending on the complexity of the tissue sample/probe combination, up to 5 PCs contribute to the original HSC to some extent.

C:

$$\mathrm{SI}_{i=\alpha-\delta}(x,y) = \sum_{\lambda} \mathrm{HSC}_{i=\alpha-\delta}\big(x,y,\lambda(i)\big)$$

$$\mathrm{SP}_{i=\alpha-\delta}(x,y) = \underset{\lambda}{\text{Spectral Position}}\Big(\mathrm{HSC}_{i=\alpha-\delta}\big(x,y,\lambda(i)\big)\Big)$$

$$\mathrm{SW}_{i=\alpha-\delta}(x,y) = \underset{\lambda}{\text{Spectral Width}}\Big(\mathrm{HSC}_{i=\alpha-\delta}\big(x,y,\lambda(i)\big)\Big)$$

where i denotes the ith spectral band (α-δ). Intra-band analysis is performed on HSC_i to achieve pixel information for each band of Spectral Intensity, Spectral Position, and Spectral Width, denoted as SI_i, SP_i, and SW_i, respectively. The calculation of SI is performed by summation using MATLAB. The calculations of SP and SW are also performed by MATLAB. To improve processing speed and make sure the calculations can be performed on general cases, peak fitting is not used. Instead, SP is calculated by finding the point of 50% accumulated area, and SW is calculated by assuming a Gaussian peak and by finding the points of 15% and 85% accumulated area (the difference is used as the SW).

D:

$$\mathrm{SI}_{i/j} = \frac{\mathrm{SI}_{i=\alpha-\delta}(x,y)}{\mathrm{SI}_{j=\alpha-\delta,j\neq i}(x,y)}$$

is utilized to characterize and maximize the image contrast based on the knowledge of the origin of contrast of each spectral region.

E: $I_{\alpha\text{-}\delta}(x, y, a, b) \rightarrow I_{\alpha\text{-}\delta}(x, y, r) : \mathrm{HDC}_{\alpha\text{-}\delta}(x, y, r)$

For each of the above-mentioned spectral bands α-δ, HDI is performed using bandpass filters. From the raw data, the directly measured results $I_{\alpha\text{-}\delta}(x, y, a, b)$ at (*e.g.* 200 × 100) positions (x, y) comprised of 320 × 256 intensity pixels (a, b) were transformed to diffuse imaging of 205 diffuse frames, $\mathrm{HDC}_{\alpha\text{-}\delta}(x, y, r)$, where r is the distance between pixel (a, b) and the centre pixel (a_c, b_c) corresponding to the incident beam location on the *XY* plane, and $r = \sqrt{(a-a_c)^2+(b-b_c)^2}$ is the radial distance from (a_c, b_c), the centre position predetermined during alignment.

F: $[\text{Coeff, Score, Explained}, \mu] = \mathrm{PCA}(\mathrm{HDC}_{\alpha\text{-}\delta}(x, y, r))$

with the functional domains for the three parameters defined as $\mathrm{Coeff}_{\alpha\text{-}\delta}(\lambda$, component = 1-3); $\mathrm{Score}_{\alpha\text{-}\delta}(x, y$, component = 1-3), which is the contrast images; and $\mathrm{Explained}_{\alpha\text{-}\delta}$(component = 1-8), which is the contribution factors. The PCA is performed by the *pca* function in MATLAB.

The first parameter, Coeff, contains information describing the transformation of PCs from diffuse frames. Coeff of the first three components is plotted to identify the most pronounced contributions from diffuse frames (**Figure S5h**). The second parameter, Score, contains the linear combination processed images for the first three PCs (**Figure S5i**), indicating the images with highest contrast obtained from the linear combination of diffuse frames. The third parameter, Explained, describes the contribution from each PC to the measured results, $\mathrm{HDC}(x, y, r)$, as shown in **Figure S5h, *inset***. The first component from PCA always dominates the HDC, and we have observed that additional information can be achieved for the second component, as discussed later in the Supporting Information.

G: $\mathrm{SR}_{i=\alpha-\delta}(x,y) = \underset{r}{\text{Scattering Radius}}\left(\mathrm{HDC}_{i=\alpha-\delta}(x,y,r)\right)$

Similar to pixel-wise analysis of HSC, pixel-wise analysis of HDC results in Diffuse Intensity and Scattering Radius information for each pixel, denoted as DI_i and SR_i. It is noted that the DI is achieved by summarizing information for all scattering distances of HDC, which is the same as the contrast image shown in **Figure S5g**. For SR, similar to SP and SW, peak fitting is not used in order to accelerate the calculation. Instead, the distance to 50% of maximum intensity is used as SR.

DOLPHIN in operation:

While using DOLPHIN for tissue or animal imaging, an area is defined first for the *X*-*Y* automated scanning stage. A typical scanning area for the tissue penetration experiments is 4 cm in length and 2 cm in width (large enough to accommodate the "MIT" shape and within the area of the tissues), and a typical scanning area for whole mouse imaging is 10 cm in length and 5 cm in width (large enough to accommodate the whole mouse). The largest area that can be scanned is 30 cm in length and 10 cm in width (large enough to accommodate a 1 kg rat). In most cases, we set the scanning resolution to achieve a final image of 200 × 100 pixels. As a result, the scanning speed is determined by the scanning length, scanning resolution, and integration time required at each pixel. For instance, for a scanning length of 10 cm, scanning resolution of 200 pixels, and integration time of 20 ms for each pixel, the scanning speed is set to 25 mm/s. And in order to scan 100 lines across 5 cm in width, the overall typical scanning time is around 400 s. Considering the acceleration and deceleration of the stage and data storage time, a typical scan concludes within 10 min. For most of the tissue and animal studies, the power of the laser illumination is less than 0.5 W/cm^2. The illumination time for each point on the scanning/imaging line is kept below 0.2 s due to the fast scan speed (this prevents any heating effect from the laser illumination), and the overall illumination time for each point on the scanning/imaging plane is estimated below 1s considering that each point is included in multiple scanning lines. For samples with complex geometry, the surface/height profiles are scanned by a 3D scanner (NextEngine). The 3D scanner is set up on top of the scanned object. During the 3D scan, a 3D point cloud of the top surface of the scanned object is achieved.

The data is stored as either a 16-bit integer or a single-precision floating point number. In the case of a 16-bit integer (single-precision floating point number), the required storage for the data of a typical experiment is 1.6 (3.2) gigabytes, with a 200 × 100 scanning resolution and 320 × 256 detector pixels. The original data collected by the InGaAs camera is stored in .spe data files and converted by MATLAB into matrix format (also stored in .mat data files) for analysis.

Home-developed software (LabView and MATLAB) allows complete automation of the laser, scanning stage, and camera control, while the basic data conversion and data analysis are conducted in real time.

Analyses of HSC and HDC for spectral and scattering features of tissue penetration

Using DOLPHIN, we performed tissue penetration studies (**Figure S5a**). Three types of rare-earth doped nanoparticles (NPs) (~ 60 nm, Figure **S14**, see pages S22-S23 for synthesis details), $NaYF_4$:Yb:Er (Er-NP), $NaYF_4$:Yb:Pr (Pr-NP), and $NaYF_4$:Yb:Ho (Ho-NP), were placed directly underneath the tissue forming the letters "M", "I", and "T" respectively (the stroke width of the letters is 1-2 mm). The rare-earth based probes are selected due to the distinct fluorescence emissions of various dopants, as well as their optical stability against air, water, illumination, and aggregation, which minimizes the variations in probe fluorescence for tissue penetration studies[1]. As for the concentration of the rare earth nanoparticles, for most of the *ex vivo* experiments, we used the nanoparticles either in pure solid form or in the form of polymer-particle blend (mass ratio 2:1, see Supporting Information for details of fabricating nanoparticle clusters of various sizes). The fluorescence signal intensity is controlled by the size of the pure solid or polymer blend. For *in vivo* experiments, a polymer-particle blend (mass ratio 2:1) is used. An area encompassing the tissue and NPs is excited by a laser of 980 nm in the trans-illumination scanning configuration, and HSC and HDC are collected and analysed.

The analyses of HSC and HDC, essentially visualization of 3D results by dimensionality reduction, are facilitated by initially recognizing the overall spatial, spectral, and scattering properties, as well as by performing principal component analysis (PCA), which further identifies important features of multidimensional data. The analyses of HSC and HDC (**Figure 4**) for penetration through a 3 cm-thick breast-mimic phantom (BioMimic optical phantom provided by INO, see Supporting Information for details) are shown as an example (**Figure S5**). The overall (or mean) spatial features (**Figures S5c, S5g**) and the overall spectral or scattering features (**Figures S5b, S5f**) are achieved by summation along relevant dimensions. For multidimensional HSC or HDC, PCA initially identifies the prominent spatial features (**Figures S5e, S5i**) with corresponding frequency or scattering coefficients (Figure **S5d,h**), and the relative contributions (***insets*** of **Figures S5d, S5h**) from each PC (PC1 represents the most highly contributing PC, and so on).

The analyses of HSC by PCA identifies groups of pixels (contrast images) with the same spectral features (PC coefficients). Excellent contrast images (**Figure S5e**) are created by linear combination of the reconstructed 2D images at different wavelengths (**Figure S4**), and the

corresponding PC coefficients in the frequency domain (**Figure S5d**) describes how 2D images from different wavelengths are linearly combined (see Supporting Information for image blurring and minimum resolvable feature sizes of the PCA-produced images). Among the PCs, different features (particularly, letters of rare-earth doped fluorescence probes and tissue in the background) are identified according to fluorescence features in the spectral domain (PC1 to PC5). PC6 and higher components show mainly noise, and contribute very little to the overall results (**Figure S5d** ***inset***). Furthermore, the frequency coefficients (**Figure S5d**) identify four distinct spectral bands, which are attributed to various light-probe-tissue interactions as follows: (1) α-band (~980 nm, PC5) represents contrast by excitation light, (2) β-band (~1100 nm, PC1,2,4) arises from small-Stokes' shift tissue autofluorescence (<2,000 cm^{-1}) and probe emission of Er-NP and Ho-NP, (3) γ-band (~1,350 nm, PC3,5) originates from large-Stokes' shift tissue autofluorescence (>2,500 cm^{-1}) and probe emission of Pr-NP, and (4) the δ-band (~1,600 nm, PC2) represents probe emission of Er-NP. It is noted that δ-band features probe emission without interfering signals. Additionally, each spectral band is characterized individually (**Figure 4a)**, by integrated spectral intensity (SI), spectral peak position (SP) and spectral peak width (SW) analyses. While SIs give information similar to applying spectral filters during conventional fluorescence imaging, small changes in SPs and SWs could potentially reveal subtle variations in the environment surrounding the fluorophores, and enhance image contrast if the probe fluorescence overlaps with tissue autofluorescence. In addition, recognizing the photo-physical origin of the spectral bands, band division processing, which compares the SI from different spectral bands, is demonstrated to be more efficient for contrast enhancing than a linear combination from PCA (**Figure S5**). In contrast to applying HSI and associated analyses in the visible and NIR-I spectral regions which silicon cameras can detect, the broad spectral span in the entire NIR region studied here (850-1,650 nm) has the advantage of a large Stokes' shift, such that α-δ bands can be easily separated (since similar Stokes' shifts result in larger excitation-emission separation in NIR-II than in visible/NIR-I wavelengths). While prior HSI technologies, in particular for remote sensing, utilize PCA-like statistics for clustering pixels[2], we explored analysis more specific to fluorescence-based HSI, e.g., SP.

Similarly, the analyses of HDC by PCA identifies groups of pixels (contrast images) with the same scattering features (PC coefficients). For HDI, optical filters are applied to measure and analyse

the scattering effect for each spectral band, β (Er-NP and Ho-NP), γ (Pr-NP), and δ (Er-NP). **Figures S5f-S5i** and **Figure 4b** show the PCA of the results obtained by applying optical filters for the δ-band. The contrast images with spatial features (**Figure S5i**) by linear combination of the reconstructed 2D images at different scattering distances (**Figure S6**), and their corresponding PC coefficients in the scattering domain (**Figure S5h**). While PC1 and PC2 show fluorescence emission from the same probes (Er-NP), they have different spatial signatures, because for PC2 the incident laser is scattered by the surrounding tissue before exciting the fluorescence probes, and for PC1 the incident laser excites the probes through a more direct path. PC3 and higher components show noises and contribute insignificantly to the overall results (**Figure S5h *inset***). Furthermore, pixel-wise scattering-profile analysis is performed to generate the scattering radius (SR) images (**Figure 4b**), as emission profiles possess cylindrical symmetry for most tissue penetration studies, due to the regular shape and relative uniformity of the tissues. The SR image describes the diffuse scattering property at each position in the imaging plane, relating to a combination of the depth of the signal source, the shape and the optical properties of the surrounding tissue. Particularly, probe emission travelling through deep tissues gives rise to a flattened photon fluent on the tissue surface (photon-exiting plane), resulting in a broadened emission contour and images without well-defined features. In contrast, by the analysis of HDC, much of the diffuse scattering of the probe emission is excluded from the resulting contrast images (by summation or other statistical analyses, such as max and median operation), and the diffuse scattering property is identified for each position to create SR images. It is noted that while SR depends on the distance travelled through tissue, the knowledge of this distance is not required in order to interpret the SR data. Instead, by interpreting the SR data (for cylindrical symmetry cases), the knowledge of distance travelled is achieved by fitting the data (see later section). In addition, since biological tissues scatter longer wavelengths to a lesser degree than shorter wavelengths, the same diffuse analysis for NIR-II fluorescence emission can resolve signals from deeper sources compared to NIR-I signals.

In summary, we have demonstrated a major advantage of DOLPHIN, which is the capability to embrace the effects of scattering and tissue autofluorescence by combining the advantages of NIR-II fluorescence imaging for deep penetration, as well as the spectra and diffuse resolving functionalities of HSI and HDI. As a result, it allows DOLPHIN to improve image contrast and

enhance our visualization for small features, compared to conventional optical imaging modalities which rely on background subtraction techniques to eliminate these effects.

In contrast, prior HSI[2,3] and HDI[4] technologies worked mainly in the visible and NIR-I regimes and utilized either epi-illumination or reflectance configurations to measure absorption, reflection and scattering of tissue properties, thus limiting the penetration depths possible (see Supporting Information for more comparison of DOLPHIN with other optical imaging technologies, including HSI, diffuse optical imaging, and NIR-II imaging). Furthermore, the information achieved through a trans-illumination configuration allows for several unique types of analyses, including principal component analysis (PCA), pixel-wise spectral and scattering analyses, as well as 3D reconstruction. As a result, over a broad spectral range of 850-1650 nm, DOLPHIN has the capability to distinguish the optical signatures of the primary pump laser, background, various types of tissue autofluorescence, and reporter fluorescence, as well as the diffuse scattering features of the fluorescence signal upon transport through heterogeneous turbid optical media.

It is noted that, the PCs (PCA basis functions) are determined numerically. Therefore, no prior knowledge of the probe fluorescence is required to obtain the PCA results. Although it is not how PCA results are typically interpreted, the basis spectra could be modified on propagation thorough biological tissues. Nonetheless, we used PCA to initially identify the information achieved by HSI and HDI, mainly through dimensionality reduction. The following analysis (e.g. spectral/diffuse analysis, depth fitting) has no direct relation to PCA.

Theoretical depth of penetration

While tabulated results from extensive measurements of tissue penetration studies allow us to empirically determine the probe depth for both HSI and HDI modes in certain special cases (*e.g.*, cylindrical symmetry of inspected tissue is required for HDI analysis), a more general approach to inferring signal depth and the optical properties of the surrounding environment is needed. Therefore, we explored the methods of theoretical calculations to determine the depth and the surrounding environment of the fluorescence signal and to reconstruct 3D images using DOLPHIN.

For HSI mode, in order to calculate the depth of the fluorescence signal at a certain spatial position (x,y), Beer's law is applied, $\ln\frac{I(\lambda)}{I_0(\lambda)} = -d \cdot \mu_{\mathrm{eff}}\,(\lambda) + constant$, where $I_0(\lambda)$, $I(\lambda)$, and $\mu_{\mathrm{eff}}(\lambda)$ are

the intrinsic fluorescence intensity of the probe at zero depth of penetration, measured fluorescence intensity through tissue, and the effective attenuation coefficient of tissue, respectively, as functions of wavelength. The signal depth d can be obtained by linearly fitting $\ln \frac{I(\lambda)}{I_0(\lambda)}$ with respect to $\mu_{\mathrm{eff}}(\lambda)$. It is noted that only the spectral shapes rather than the absolute values of $I(\lambda)$ and $I_0(\lambda)$ are required for the linear fitting. **Figures S13a-S13c** show the measured emission spectra of Er-1575 band from HSI and estimated $\mu_{\mathrm{eff}}(\lambda)$ of a breast-mimic phantom[5–10]. The fitted results for tissue penetration at depths of up to 20 mm match well with the actual depths (**Figure S13d**), indicating the effectiveness of calculating depth from HSI mode. It is noted that calculating signal depth from HSI mode would only be effective in the case that a sufficient level of emission signals could be achieved for a range of wavelengths with different μ_{eff}, (*e.g.*, 1100–1200 nm, 1500–1600 nm). We believe that the combination of a deep-penetrating NIR-II fluorescent signal and HSI mode makes it feasible to perform this depth profile calculation.

For calculating depth and μ_{eff} from HDI mode, we first considered the case in which the scattering profile possesses cylindrical symmetry, $I(r)$, for regularly shaped and uniform tissues (Figure **S9e,f**). Assuming the emitted light travels from depth d as a spherical wave in a homogeneous optical medium with μ_{eff}, the equation: $I(r)\cdot(r^2+d^2)\cdot e^{u_{\mathrm{eff}}\cdot\sqrt{r^2+d^2}} = constant$ is used to fit both depth d and μ_{eff}. The fitted results for d and μ_{eff} match well with the actual depth and estimated μ_{eff} values, respectively, for various tissues[5–10] (**Figures S13f, S13i-S13m** and **Figure 6**). Furthermore, for a general case without a cylindrically symmetric scattering profile $I(a, b)$ due to irregular shape of the tissue or animal (**Figure S13g**), a similar relation can be used to calculate the probe depth by (1) assuming the tissue is a homogeneous optical medium and (2) using the height/surface profile of the subject obtained by a 3D scanner. Consequently, the fitting equation changes to: $I(a,b)\cdot(r^2+h^2)\cdot e^{u_{\mathrm{eff}}\cdot\sqrt{r^2+h^2}} = constant$, where $r^2=(a-a_0)^2+(b-b_0)^2, h=d-z(a_0,b_0)+z(a,b)$; a and b are the in-plane spatial coordinates; z is the height at each location of (a, b); and a_0 and b_0 are the centre location coordinates of the incident beam. Combining the fluorescence signal $I(a, b)$ and the 3D scanned height profile $z(a, b)$, both depth d and μ_{eff} can be fitted (**Figure S13h**). The fitted results of depth d are in agreement with the actual values (which can be seen in the 3D reconstruction, **Figures 7a-7f**), though the residuals of the fitness are larger than for the cylindrical homogeneous cases mainly due to the simplifying

assumption of homogeneity for a heterogeneous subject, in particular the animal. It is worth reiterating here, that the real case of a tissue sample or whole animal is quite heterogeneous, similar to the data presented in **Figures S13g, h**, and different compared to the phantom data shown in the idealized proof-of-concept in **Figures S13e, f**. Therefore, we have represented the fitted depth as a curve in **Figure S13f**, for the tissue phantom exhibiting cylindrical symmetry; while the fitting results are shown as a solid surface profile in **Figure S13h** for the mouse data. In other words, **Figure S13f** is only a special case of **Figure S13h**, where radial symmetry is present (in the former).

Overall, it is demonstrated that signal depth can be derived from both HSI and HDI. While HSI has the advantage of identifying autofluorescence, HDI offers more accurate results of fitted depth for a large variety of tissues without knowledge of the tissue type. Additionally, HDI predicts μ_{eff} sufficiently close to the estimated values with the scattering profiles as the only information, which presents an opportunity to identify and distinguish different types of tissues. To the best of our knowledge, this is the first demonstration to combine deep-penetrating NIR-II fluorescence and HDI mode to fit penetration depth and tissue properties. Notably, the trans-illumination configuration employed in the DOLPHIN imaging system further benefits the depth analysis of both HSI and HDI, compared to systems based on epi-illumination (**Figure 6b**), due to further lowered excitation scattering and autofluorescence background (which is already reduced for NIR-II fluorescence compared to visible or NIR-I fluorescence) from the surface of the tissue.

The current DOLPHIN system has certain limitations to predicting the depth of fluorescent probes. First, HSI is less practical for predicting the depth of penetration in a complex system, like an animal, likely due to the unknown value of the heterogeneous μ_{eff} in real animals. On the other hand, the presence of multiple sources of fluorescence, with different brightness and at different depths in the tissue, can easily be detected in HSI mode. This, however, is beyond the scope of the present work, and remains to be validated as an area for future investigation with the DOLPHIN system. The relative intensities of $I_0(\lambda)$ of rare earth-based NPs are rarely affected by dosage or the local environment due to f-electron transition-induced fluorescence. Even though the dosage changes the absolute value of $I_0(\lambda)$, it does not affect the fitting, since the fitting equation has a constant parameter, which makes the relative intensities of the $I_0(\lambda)$ the only factor that needs to be considered. Second, as we perform a 2-D scan and if there were two or more patterns of features on top of each other inside the tissue, it would be nearly impossible to predict at which depth the

signal is coming from. As a result, the current imaging method and algorithm would attempt to retrieve the "weighted average" of the two depths. We believe that this limitation of the current system could be overcome by a cylindrical coordination scan, scanning at least half of the entire surface area of tissue, which is similar to the current MRI and CT scanning schemes. On the other hand, if the two or more patterns at different depths are not located exactly on top of each other (separated by a distance greater than the scanning resolution), the current system should be able to distinguish the patterns and resolve the depth information separately.

Theoretical estimation of minimum number of labelled cells required for detection *in vivo*

Our calculations show that DOLPHIN provides the possibility of few cell imaging in mouse models. For this, we assume the following: (a) the incident power density of the excitation source (laser) to be 100 mW/cm^2, and (b) an uptake of 1000 RENC NPs per cell (10% labeling efficiency, which is conservative according to FACS cell sorting data, not shown here, upon incubation with 10,000 NPs per cell; typically, we obtain labeling efficiencies >> 20% of incubated NPs), see Fig. S15 for example.

To calculate the absorption efficiency (γ) of each rare earth-doped NP, we first begin with a Beer-Lambert form of the absorbed intensity: $\frac{I}{I_0} = e^{-\sigma \cdot n \cdot l}$, where I_0 is the incident power, I is the transmitted power after being absorbed through the NP, with n being the density of absorbing ions (Yb^{3+}, for rare earth-doped NPs used in the present study), σ being the absorption cross-section of each ion, and l being the path length (equal to the diameter of the NP, in this case, 60 nm). We first need to estimate the density of ions, n. Based on values reported in the literature[11], the molecular weight of a 60 nm particle of $NaYF_4$:Yb:Er (in 78:20:2 at.%) is 320 MDa. A single unit cell of this crystal ($NaY_{0.78}F_4Yb_{0.2}Er_{0.02}$) has a formula weight of 206.3 Da. Taking into consideration the doping fraction of 0.2 for Yb^{3+}, the volume density (n) of Yb^{3+} ions in a nanoparticle with diameter 60 nm, therefore can be estimated to be: $n = \frac{0.2 \times 320 \times 10^6}{206.3 \times \left(\frac{4}{3}\pi(30 \times 10^{-7})^3\right)} = 2.74 \times 10^{21}$ cm^{-3}. For a single Yb^{3+} ion, the absorption cross-section value reported in the literature[12] is: $\sigma = 1.17 \times 10^{-20}$ cm^2. Therefore, the transmitted power is $I/I_0 = e^{(-1.17 \times 10^{-20} \times 2.74 \times 10^{21} \times 60 \times 10^{-7})} \approx 0.9998$, resulting in an absorption efficiency: $\gamma = 1 - \frac{I}{I_0} = 0.0002$.

Taking an average NP size of 60 nm used in this study, the excitation power incident upon each NP, as: $P_{\mathrm{ex}} = \pi(30 \times 10^{-7})^2 \times 0.1 = 2.83$ pW per NP. Then, using a quantum yield of 1.1% for the down-conversion process of Er-doped NP, the emission power per NP is: $P_{\mathrm{em}} = 2.83 \times 10^{-12} \times 0.0002 \times 0.011 = 6.23 \times 10^{-18}$ W. While the classical approach to calculating the emission power (P_{em}) for a single particle, one-step transition is based on the absorption cross-section (σ) of a single ionic species, estimated as $P_{\mathrm{em}} = \eta_{\mathrm{QY}} \cdot \sigma \cdot P_{\mathrm{ex}}$, where η_{QY} is the quantum yield of the emission process, P_{ex} is the excitation power incident on the emitter, it is worth noting that the calculation here is performed based on the geometry of an ensemble of absorption centers (Yb^{3+} sensitizers) per NP, as reported in the literature[13], in the form of $P_{\mathrm{em}} = \eta_{\mathrm{QY}} \cdot \gamma \cdot A \cdot P_{\mathrm{ex}}$, where the absorption cross-section of an individual emitter ion (σ) is replaced by the factor γA, where $A = \pi R^2$ is the area of cross-section of a spherical NP of radius R, and γ is the geometric efficiency factor of absorption, calculated above using the Beer-Lambert form of expression. Based on our earlier assumption of 1000 NPs uptake per cell, the average emission power per cell is, $P_{\mathrm{em,cell}} = 6.23 \times 10^{-18} \times 1000 = 6.23$ fW. To get an estimate of the number of photons emitted, we use the emission wavelength of Er-doped NPs: λ_{em} = 1,575 nm. Using the energy produced per photon, $E = hc/\lambda$ (where h is Planck's constant and c is the velocity of light in vacuum), we can estimate the total number of photons emitted per cell (per second) to be:

$$N_{\text{photons emitted, per cell, per second}} = \frac{P_{\text{em, cell}}}{hc/\lambda_{\mathrm{em}}} = \frac{6.23 \times 10^{-15}}{(6.626 \times 10^{-34} \times 3 \times 10^{8})/(1575 \times 10^{-9})} = 4.94 \times 10^{4}.$$

These photons, when emitted from a fluorophore deep inside body tissue, undergo absorption and scattering events as they travel through a certain depth of tissue and are ejected at the surface, to be picked up by the InGaAs detector of our imaging camera. Using the attenuation coefficient of body tissue to be $\mu_{\mathrm{eff}} \sim 0.9$ cm^{-1}, for a body tissue depth of 2 cm (the typical depth of a mouse body), the number of photons reaching the surface:

$$N_{\text{photons ejected at surface of tissue per cell (2 cm depth)}} = 4.94 \times 10^{4} \times e^{(-0.9 \times 2)} = 8.16 \times 10^{3}$$

photons per second, from each labeled cell.

From the surface, only a fraction of the photons reaches the detector sensor. We assume the surface of the tissue subtends 5% of the total solid angle (4π) at the detector. Further, according to the specifications of the 2-D OMA V: 320-1.7 LN InGaAs detector (Princeton Instruments), the sensor has a Quantum Efficiency of > 80%. Therefore, out of the photons reaching the surface of the

tissue (from 2 cm depth) per second, the total photons collected at the detector (per labeled cell) is: $N_{\text{photons reaching detector,per labeled cell (2 cm depth)}} = 8.16 \times 10^3 \times 0.05 \times 0.8 = 3.26 \times 10^2$.

For HSI mode of imaging, these photons will be detected over the 256 pixels of the sensor (the other dimension of 320 pixels is used for wavelength spectral analysis). Therefore, for a single labeled cell, the number of photons collected, per pixel, per second is: $N_{\text{photons collected at detector, per pixel, per second}} = \frac{3.26 \times 10^2}{256} = 1.27$ photons per pixel, per second, reaching the detector, per labeled cell, from a depth of 2 cm in tissue.

Next, we estimate the minimum number of photons required for reliable detection, for a signal-to-noise ratio of 3. The total noise can be estimated as: $\text{Noise}_{\text{total}} = \sqrt{(S + DCR + RN^2)}$, where S is the fluorescence signal, DCR is the dark current readout (5000 e^-/pixel/sec) and RN is the readout noise, which is RN_L = 1500 e^- rms (low gain) and RN_H = 50 e^- rms (high gain). For weak signals: we would like to have at least S/N = 3 for reliable detection, or $S \geq 3 \cdot \text{Noise}_{\text{total}} = 3\sqrt{(S + DCR + RN^2)}$. Using the high gain scenario (DOLPHIN operates the sensor in high gain), solving the above quadratic expression we get $S \approx 265$ e^-. In other words, to achieve a minimum signal-to-noise ratio of 3, the required signal level is S = 265 e^-/pixel.

Finally, to achieve a signal level of 265 electrons per pixel, the minimum number of labeled cells required for reliable detection, from a depth of 2 cm in tissue, is:

$$N_{\text{minimum threshold of labeled cells for reliable detection } (S/N = 3)} = {}^{265}/_{1.27} = 207.9 \sim 208$$

We perform the same estimation for the scenarios of 0, 6 cm and 8 cm depths through tissue, which are tabulated below. In summary, we should be able to **realistically detect a tiny cluster of hundreds of cells through the body of a whole mouse, or a tiny sub-millimeter tumor through up to 8 cm in breast tissue** (assuming a mammalian cell size of ~ 20 µm). Therefore, we believe that DOLPHIN opens up the possibility of detecting signal from few-cell clusters at depths up to 2 cm, and from sub-millimeter sized tumors through 6-8 cm of breast tissue or breast-mimic phantom. The following table summarizes the minimum threshold of detection for various types of imaging configurations using the DOLPHIN system.

Depth of Imaging	Type of Tissue (Attenuation coefficient, in cm^{-1})	Min. # of cells required (labeled with 1000 NPs/cell)
0 cm	No phantom or tissue intercalated between fluorophore and detector	35
2 cm	Breast-mimic Tissue Phantom / Human Breast Tissue (0.9 cm^{-1})	208
6 cm	Breast-mimic Tissue Phantom / Human Breast Tissue (0.9 cm^{-1})	7607 (approx. tumor size of 0.4 mm)
8 cm	Breast-mimic Tissue Phantom / Human Breast Tissue (0.9 cm^{-1})	46020 (approx. tumor size of 0.72 mm)

How realistic is the premise of labelling 1,000 NPs per cell? For a mammalian cell with a diameter of ~ 20 µm, the ensemble of 1,000 NPs each with a diameter of 60 nm would occupy a volume fraction: $\frac{1000 \times \frac{4}{3}\pi(30\times10^{-9})^3}{\frac{4}{3}\pi(10\times10^{-6})^3} = 0.000027$, or < 0.003%. Therefore, it is reasonable to expect that even with an internalization of 1,000 NPs per cell, the volume is not large enough to disrupt intracellular processes or otherwise lead to toxicity.

It is worth noting, that the calculation performed here based on the assumption of all the excitation power (100 mW/cm^2) of the laser reaching the fluorophore to be excited (similar to what is depicted in the schematic in Fig. S5(a), where the laser is directly incident on the “M”, “I” and “T” fluorophores, and only the emission signal has to travel through the tissue or phantom). In reality, however, for fluorophores buried deeper in tissue, in addition to the emission signal, the excitation light will also need to travel a certain distance through tissue before encountering the emitter, and this will result in scattering losses of the excitation source, requiring a higher power density of the laser source (or a larger number of fluorophores emitting), which could result in the requirement of a greater minimum threshold of labelled cells for detection, at all depths involved. We also note here that the maximum permissible exposure of skin to laser irradiation at 980 nm (calculated as per Table 7 of the ANSI Z136.1-2007 standard, in the wavelength range 700 - 1400 nm for CW lasers) is ~ 0.7 W/cm^2 (for exposures up to 10s, which is more than the integration time at any point of the raster scan method used in DOLPHIN). Therefore, there is a possibility of safely increasing the incident laser power beyond the 100 mW/cm^2 used in the present work (up to a factor of 5×), to partially compensate for the scattering and absorption losses of the incident light as it travels through tissue.

Theoretical prediction of maximum depth of detection of 1 mm-sized cluster probe in breast tissue with NIR-II light

We decided to perform a theoretical calculation to predict the limit of the maximum depth of penetration using the propagation of NIR-II light, using some of the experimental parameters which are relevant to the DOLPHIN setup described in this manuscript, as follows:

For a 1 mm-sized cluster probe (comprised of an ensemble of 60 nm nanoparticles): the absorption cross-section is: $\pi \times (0.5 \times 10^{-3})^2 = 7.85 \times 10^{-7}$ m^2. Given the input laser excitation power density of 100 mW/cm^2, the power incident on the 1 mm cluster probe is: $P_{\mathrm{input}} = 7.85 \times 10^{-3}$ cm^2 $\times$ 0.1 W cm^{-2} = 0.785 mW.

Then, using the absorption efficiency $\gamma = 0.0002$ (calculated in the previous section for a single NP), and a quantum yield of 1.1%, the emission power per 1 mm cluster:

$P_{\mathrm{emission\ per\ 1mm\ cluster}} = P_{input} \cdot \gamma \cdot \eta_{QY} = 7.85 \times 10^{-4} \times 0.0002 \times 0.011 = 1.73 \times 10^{-9}$W.

To estimate the number of photons emitted by the cluster, at the emission wavelength of 1,575 nm, $N_{\mathrm{photons\ emitted\ by\ 1\ mm\ cluster}} \cdot hc/\lambda_{\mathrm{em}} = 1.73 \times 10^{-9}$ W, which gives:

$N_{\mathrm{photons\ emitted\ by\ 1\ mm\ cluster}} = 1.37 \times 10^{10}$ photons emitted per second by the 1 mm cluster of NPs.

Minimum required for detection = 265 photons/pixel at detector (based on the noise calculation in the previous section, to achieve $S/N \geq 3$)

$$265 = 1.73 \times 10^{10} \times e^{(-0.9\,d)}$$

where d is the maximum depth of penetration

Solving for d gives d = 20 cm

However, the above d_{max} was based on a single pixel detection, without any losses in collection efficiency. For HSI mode of imaging, we have to account for the spread of the photons across the 256-pixel dimension, and also account for the geometric efficiency of collection (assuming 5% of the solid angle subtended at the detector), and the Quantum Efficiency of the detector (80%). Therefore, the minimum number of photons required for reliable detection, to achieve $S/N \geq 3$, is:

$$265 \times 256 = 1.73 \times 10^{10} \times 0.05 \times 0.8 \times e^{(-0.9\, d_{\max})}$$

resulting in d_{max} = 10.26 cm. Therefore, our results of the maximum depth of detection (~ 7 cm in phantom using HSI mode of imaging, see Figure 6 in the main text) is well within the limits of the theoretical maximum depth of detection.

Theoretical estimation of maximum depth of detection of a fluorescently-labeled 1 mm-sized tumor in breast tissue *in vivo*

We next estimate the maximum depth of detection of a 1 mm-sized tumor (a more realistic scenario), with a reasonable assumption of labeling efficiency of 1000 NPs per cell. From the calculation in the previous section (refer to page S15), each cell emits a power of $P_{\text{em, cell}} = 6.23 \times 10^{-15}$ W, or $N_{\text{photons emitted, per cell, per second}} = 4.94 \times 10^{4}$ photons.

Consider a 1 mm-sized tumor, consisting of mammalian cells each with a diameter of 20 µm. The number of cells in the tumor can be estimated to be: $N_{\text{cells, in 1mm sized tumor}} = \frac{\frac{4}{3}\pi(0.5 \times 10^{-3})^3}{\frac{4}{3}\pi(10 \times 10^{-6})^3} = 1.25 \times 10^{5}$ cells. Therefore, the total number of photons emitted by a 1 mm-sized tumor will be:

$N_{\text{photons emitted by 1 mm-sized tumor}} = 4.94 \times 10^{4} \times 1.25 \times 10^{5} = 6.18 \times 10^{9}$ photons/second.

In this more realistic scenario, to achieve the maximum depth of detection of such a 1 mm-sized tumor, we use the minimum number of photons required (265) to achieve $S/N \geq 3$, spread over the entire 320 × 256 pixels of the InGaAs sensor in HDI mode, and also account for the geometric efficiency of collection (assuming 5% of the solid angle subtended at the detector), and the Quantum Efficiency of the detector (80%):

$$265 \times 320 \times 256 = 6.18 \times 10^{9} \times 0.05 \times 0.8 \times e^{(-0.9 \times d_{\max})}$$

which gives a maximum depth of detection, d_{max} = 2.7 cm, of a 1 mm-sized fluorescently-labeled tumor *in vivo*.

We again note here, as discussed in detail on page S17, that this calculation is based on the assumption of 100 mW/cm^2 of the laser light incident upon the fluorophore (in this case, the 1

mm-sized tumor, with fluorescently-labeled cells). In practice, this number could go lower, since the laser excitation source could undergo absorption and scattering effects in tissue, before it interacts with the tumor. However, as noted in the discussion on page S17, there is the possibility of raising the laser excitation power to compensate for these losses in the excitation source.

Raman signal as tissue autofluorescence

Biological tissues containing lipids and other chemicals scatter inelastically with strong Raman intensities at Raman shifts of ~3,000; 2,800-3,000; 1,440; and 1,300 cm^{-1}, corresponding to the unsaturated =C–H bond stretch, the saturated $–CH_2$ asymmetric and symmetric stretches, $–CH_2$ bend, and $–CH_2$ twist vibrations, respectively[14]. Therefore, for a 980 nm excitation source, it is beneficial if the probe emission is not centred close to either ~1,388 nm or ~1,135 nm, in order to mitigate the background. UCNPs such as $NaYF_4$ co-doped with Yb and Er are well-suited for these criteria, with a peak emission at 1,560 nm. This rationale is in agreement with numerous reports on utilizing the large spectral separation between excitation and emission wavelengths for biomedical imaging. However, in this work, we demonstrated that HSI of DOLPHIN can distinguish between probe fluorescence and tissue autofluorescence in both the spectral and spatial domains with PCA.

We believe that the autofluorescence signal we detected is mainly from Raman processes instead of fluorescence emission from biomolecules (e.g., flavins, lipofucins, and reticulin fibers)[15]. First, the amount that biological tissues contribute to the detected autofluorescence is much more than the fluorescent probes in the actual measured systems (depending on the amount of fluorescent probes available, the 10^{10} times difference between cross sections of Raman and fluorescence processes could be offset). Second, we found that the autofluorescence signal shifts as the excitation wavelength and the energy difference (Stokes' shift) between the excitation wavelength and autofluorescence signal remains unchanged, suggesting the Raman origin of the autofluorescence signal. Third, in order to detect the signal in the broad wavelength range from 900 to 1700 nm, we used a reflective grating with 150 lines/mm, which would not resolve the sharp Raman peaks. In addition, we speculate that there are many types of biological materials and chemical bonds that contribute to the two main regions of autofluorescence signals, which could lead to the broad peaks we observed.

Autofluorescence in NIR-II wavelengths:

Although visible and NIR-I wavelengths excite more tissue autofluorescence than NIR-II wavelengths, a degree of tissue autofluorescence always exists, no matter what the excitation wavelength is. Therefore, tissue autofluorescence in NIR-II wavelengths still affects the imaging, as noise always becomes a more prominent problem when the signal level decreases. This fact is indicated by the results in the main text and several works. Additionally, whether the tissue autofluorescence is "relatively" strong or not depends on the actual fluorescence signal intensity from the fluorescent probes used.

Image blurring and minimum resolvable feature sizes

For images in **Figure S5** and **Figure 4**, the image blurring and the minimum resolvable feature sizes depend on many factors, including signal intensity, type of tissue, and thickness of tissue. Overall, if the detected signal is strong enough, the minimum resolvable feature size should be determined by the pixel size of the processed images, which then depends on the scanning length and scanning speed.

Statistics in Figure 5:

In **Figure 5**, the mean and standard deviation for each data point are calculated from more than 10 different pixels. In fact, only when the measurements reach the detection limit, does the number of pixels available for calculation become this small. For most of the measurements (with penetration depth smaller than the limit), a relatively large number of pixels are available for calculation (the typical value is several hundreds of pixels from each measurement). If we perform measurements for independent samples, the variation mainly comes from the variation of the tissue samples (not in the case of tissue phantoms, but for real tissue). However, if the same tissue samples are used, the variation between measurements becomes similar to the variation within each measurement.

Comparing DOLPHIN to similar technologies, including other biomedical HSI, HDI, and NIR-II imaging systems:

First, HSI (in NIR-II and many other regions) is a fairly mature field. However, it is applied mostly to remote sensing and materials inspection industries, rather than to biomedical imaging applications. While there have been some applications of HSI for clinical diagnostics[3], these have mostly utilized the visible or NIR-I wavelengths due to the more well-established instrumentation. The available literature on NIR-II hyperspectral imaging has been limited to either surface-level visualization[16–19] or as an intraoperative imaging tool[20,21], with most systems implemented in reflectance mode imaging[3], with apparently no whole-body deep imaging systems available. Second, technologies similar to HDI (*i.e.*, diffuse optical imaging, DOI and diffuse optical tomography, DOT) have not been used with NIR-II wavelengths, which clearly demonstrate deep penetration and less scattering (*e.g.*, making the data analysis easier and more reliable). Third, we consider rare-earth based materials as a model system, and our technology can apply to any other NIR-II fluorescent probe materials. The reason we chose rare-earth based materials is their fluorescence properties are minimally affected by chemical or biological environments due to f-electron transition-based fluorescence. In addition, none of the previously published results using any of these NIR-II fluorescent probes applied them to either HSI or HDI technologies. However, with the unique advantage of NIR-II fluorescence (low attenuation, low scattering, and deep penetration), our method provides an apparent advantage over other methods using conventional epi-fluorescence imaging techniques.

Comparing trans-illumination and epi-fluorescence configurations:

We believe that a trans-illumination-based imaging technology (utilized by DOLPHIN) can potentially be applied to *in vivo* large animal and human imaging (upon FDA approval). As indicated in the main text and suggested by many others, a trans-illumination configuration can detect signals from deeper penetration depths than an epi-fluorescence configuration. More precisely, the detection depth for both trans-illumination and epi-fluorescence configurations is limited by the detection limit of the signal intensity (whose factors include detector sensitivity, fluorescence quantum yield, etc.) and the noise level (*e.g.*, autofluorescence), whereas the detection depth for an epi-fluorescence configuration is ultimately limited by its much higher noise level. Therefore, we propose that to perform trans-illumination imaging in humans, which still

requires higher quantum yield of fluorescence probes and more sensitive detectors, both probe and detector requirements are particularly important and potentially achievable considering the relatively short period of time that NIR-II imaging technology has been available compared to silicon camera technology.

Comparing DOLPHIN to fluorescence molecular tomography (FMT):

Fluorescence molecular tomography (FMT) aims to generate a 3D reconstruction of the distribution of molecular probes in tissues, based on light measurements at the tissue boundary or outer surface. This 3D reconstruction is based on illumination of the tissue of interest at various points (or solid angles of projection), with the collected light processed by a mathematical formulation describing the propagation of photons in a scattering medium[22]. There are three types of illumination sources available for the design of optical imaging systems: continuous wave (CW), which uses light of constant intensity; time-domain (TD), which uses ultra-fast pulsed excitation, with pulse widths ~100 fs to 100 ps; and frequency-domain (FD), which uses light modulated at frequencies ~100 Mhz to 1 GHz. For biological imaging purposes, most FMT systems usually rely on continuous wave (CW) excitation sources, in order to achieve deeper photon penetration through measurement of the change in light attenuation, as well as a better SNR, along with the advantages of simpler, lower-cost instrumentation and ease of operation. However, CW excitation leads to lower spatial resolution compared to other types of excitation, such as time-domain (TD) or frequency-domain (FD) modes, as well as the lack of ability to study ultrafast kinetic process such as the fluorescence lifetime of a fluorophore. In contrast, DOLPHIN combines the simplicity of CW technology with a heuristic approach to 3D reconstruction, which uses spectrally-derived parameters (SP, SW and SR) to obtain spatial information (**Figure 5** of the main manuscript, **Figure S13(h)**), thus avoiding a loss of spatial resolution.

In terms of instrumentation design, two kinds of FMT systems are used: contact and non-contact systems. In contact-based systems, the detectors (optic fibres) are placed in contact, *i.e.*, coupled directly, with the subject being imaged through the use of a matching fluid. In non-contact or free-space fluorescence tomography systems, 360° imaging is made possible using a CCD camera, by capturing projections of the object from various angles in a trans-illumination configuration, with the specimen being rotated and the CCD camera located opposite to the excitation source. While there is a general trend to move away from contact-based systems (to avoid the use of fluid media),

even for non-contact systems, the need for multiple source-detector configurations or for rotation of the specimen adds to instrument complexity. DOLPHIN overcomes these challenges by using a fixed camera and fixed excitation source, while rastering imaging subject positions using a motorized system in a trans-illumination configuration across the specimen stage, to cover the entire region of interest.

The typical limit of detection for CW FMT systems is 1–3 mm at depths of ~2 cm in contact-based systems[23], to approximately 1 mm at a depth of 1.5 cm in the best implementations[24]. In contrast, our DOLPHIN system offers resolutions of ~100 µm at a depth of 2 cm (through the whole-body thickness of a mouse) and ~1 mm at a depth of 4 cm (through the whole-body thickness of a rat).

In conventional FMT imaging systems, there are two methods used to deal with optical heterogeneity in the tissue medium: iterative numerical solutions to the coupled diffusion equations[25] (calculating photon attenuation in tissues, and then using it to solve the inversion problem in the forward model of the diffusion phenomenon), or minimizing the sensitivity of background optical heterogeneity by using the normalized Born approximation[26]. For both approaches, there is a need for an *a priori* calibration procedure, in order to estimate the background absorption value for baseline measurements. This may not always be practical or feasible, in the case of living subjects. In contrast, the DOLPHIN system relies on a heuristic approach to determining the depth and anatomical location of fluorophores, using a two-step process (assuming constant optical properties of the tissue medium, in a first-order approximation neglecting tissue heterogeneity). In the first step, HSI is performed to identify spectral regions of interest through PCA. In the second step, HDI is performed and overlaid with surface reconstructions of the anatomy, to get an estimate of the depth through the scattering radius, without the need for calibration or *a priori* knowledge of the absorption characteristics of the tissue.

It should be noted that 3D diffuse optical tomography has been performed in a small-scale study in human breast imaging[27], using indocyanine green injection and a trans-illumination imaging modality. Therefore, it is reasonable that our DOLPHIN imaging system can be suitably adapted for trans-illumination imaging of human subjects. Compared to epi-fluorescence (reflectance) imaging, trans-illumination is believed to provide light-tissue interaction information throughout

the whole depth of the subject (not merely sub-surface interactions), and is therefore better suited[28] to gathering quantitative data about the depth and concentration of the fluorophores.

Synthesis of rare-earth doped nanoparticle probes:

The synthesis of rare-earth doped nanoparticle probes ($NaYF_4$:Yb:X = 78:20:2 atomic %, X = Er, Ho or Pr) is adapted from a published work[29]. All syntheses were carried out using standard Schlenk techniques. 1-Octadecene (ODE; technical grade, 90%), oleic acid (OA; technical grade, 90%), and $Na(CF_3COO)$ were purchased from Sigma Aldrich. $RE(CF_3COO)_3$ (RE=Y, Yb, Er, Ho, Pr) was purchased from Rare Earth Products, Inc. A typical protocol for the synthesis of hexagonal phase $NaYF_4$-based down-conversion NPs (DCNPs) is described as follows, for obtaining 2 millimoles (final yield) of the Er-doped NPs (and similarly for the Ho-doped or Pr-doped NPs):

**Name of Chemical**	**Chemical Formula**	**Molecular weight (gm)**	**# of moles (in mmoles)**	**Weight (in mg)**
Sodium trifluoroacetate (in 2× excess)	$Na(CF_3COO)$	136.02	4	544.04
Yttrium trifluoroacetate (trihydrate)	$Y(CF_3COO)\cdot 3H_2O$	481.95	1.56	751.84
Ytterbium trifluoroacetate (trihydrate)	$Yb(CF_3COO)\cdot 3H_2O$	566.09	0.4	226.44
Erbium trifluoroacetate (trihydrate)	$Er(CF_3COO)\cdot 3H_2O$	560.03	0.04	22.41
**Yield**	$NaYF_4$:Yb:Er (78% Y, 20% Yb, 2% Er)	206.30	2	412.6

A certain amount of $Na(CF_3COO)$ and $RE(CF_3COO)_3$ (see Table above for the exact amounts) were added to a three-necked flask and mixed together with 5 mL of ODE and 5 mL of OA. The mixture was then heated under vacuum at 100 °C for 45 min to form a transparent, yellow solution. In another three-necked flask, 15 mL of ODE and 15 mL of OA were added, and flushed with argon for 15 min, then heated to boiling, at ~ 330 °C. The preheated precursor is then added into the flask with boiling solvent, where a large amount of white smoke was produced, indicating the decomposition of metal trifluoroacetates. After 20-35 min of reaction under argon flow and vigorous magnetic stirring, the solution was cooled down by adding 15 mL of ODE. The products were isolated by adding 50 mL ethanol followed by centrifugation at 3200 rpm for 5 min. Due to the monodispersity of the as-synthesized samples, no size-selective fractionation is needed. The DCNPs were redispersed in chloroform with a nanocrystal concentration of about 20 mg/mL.

Preparation of various aggregate sizes of Er-NP and Ho-NP:

Polyethylene glycol (PEG, 20 kDa), polycaprolactone (PCL, 80 kDa), poly-L-lactide (PLA, 50 kDa), and polyvinyl alcohol (PVA, 75 kDa) are purchased from Sigma-Aldrich.

Different sizes (1 mm, 100 μm, and 10 μm) of aggregates of Er-NP and Ho-NP are used for tissue and whole animal penetration depth studies, as shown in **Figure 6b** and **Figure 7** in the main manuscript. For 1 mm and 100 μm sized aggregates, Er-NP or Ho-NP is mixed with polymer (PEG, PLA, or PCL) at 1:2 mass ratio. Chloroform is added as solvent to the mixture to dissolve the polymer and disperse the NPs. After the solvent is evaporated, a uniform composite of NPs embedded in polymer is formed. Upon mild heating, the NP-polymer composite can be softened and deformed, and thus moulded into different sizes or shapes. Then the composite is typically stretched or cut into small pieces, from which aggregates of 1-2 mm and 100-200 μm are identified by eye or microscope and used for the penetration studies.

For the 10 μm sized aggregates, PCL (100 mg) and DCNPs (200 μL, 20 mg/mL) are mixed in chloroform (2 mL), followed by sonication at room temperature for 5 min to give a PCL/DCNP blend solution. PVA (1 g) was dissolved in 100 mL distilled water and stirred with a magnetic stirrer at 70 ºC for 24 hr until completely dissolved. The solution was then cooled to room temperature. The PCL/DCNP solution (2 mL in chloroform) was added to the PVA solution (1%, 100 mL) and stirred at 500 rpm for 3 hr to allow for complete evaporation of chloroform. The produced microspheres were collected by filtration, washed with distilled water, and diluted to the desired concentration, such that by applying this concentration of microspheres, one or two particles approximately 10 μm in diameter could be seen under bright-field microscope.

Characterization of NPs:

For transmission electron microscopy (TEM), 5-10 μL of NP solution is drop-cast onto carbon-coated Cu grids. TEM images are acquired by FEI Tecnai (120 kV) or JEOL 2010 (200 kV).

Fluorescence spectra are measured by a NanoLog spectrofluorometer (Horiba Jobin Yvon). The excitation is provided by a 980 nm laser. The NIR-II fluorescence signal through a monochromator (spectral resolution of 5 nm) is collected by an InGaAs detector, which is cooled by liquid nitrogen to reduce noise from the detector, except for Er-NPs, which emits above 1,600 nm and cannot be detected while the InGaAs detector is cooled (the detector is thus kept at room temperature).

Fluorescence quantum yields are measured by a home-built setup with an integrating sphere, where the samples are placed[30]. The integrating sphere used in our study has 2 open windows: one for sending in the excitation light, and one for collecting the emission signal out of the sphere. The excitation is provided by a 980 nm laser. By putting a laser notch filter in front of the exit window, we can easily remove the excitation light, and measure the total emission quantum yield (up-conversion + down-conversion) by a digital power meter. Further, we can insert an IR long-pass filter or IR short-pass filter, as desired, to independently measure the quantum yield of down-conversion (the NIR-II emission, which is a 1-photon absorption process), or the quantum yield of up-conversion (the visible emission, which is a 2-photon absorption process), respectively. For the purposes of the DOLPHIN study, the NIR-II fluorescence signal sent through a monochromator is collected by an InGaAs detector. The three types of rare-earth doped nanoparticles (NPs) (~ 60 nm, **Figure S14**) have the following characteristics: $NaYF_4$:Yb:Er (Er-NP; Y:Yb:Er = 78:20:2; with emission peaks at 1,125 and 1,575 nm; quantum yield = 1.1% for down-conversion), $NaYF_4$:Yb:Pr (Pr-NP; Y:Yb:Pr = 79:20:1; 1,375 nm; quantum yield = 0.002% for down-conversion), and $NaYF_4$:Yb:Ho (Ho-NP; Y:Yb:Ho = 79:20:1; 1,175 nm; quantum yield = 0.02% for down-conversion). We note here that the QY measured for our Er-, Pr- and Ho-doped NPs compares with that for similar rare earth-doped nanoparticles used in the literature[31]. It is also worth mentioning, that, while the NPs used in this study also have up-conversion emission in the visible range (500 - 600 nm) of the wavelength spectrum[32], we are primarily concerned with the fluorescence signal provided by the down-conversion process, due to the advantages offered by imaging in the NIR regime[33], as discussed in the Introduction section of the main text.

Cell imaging study:

Briefly, RAW 264.7 (ATCC) murine macrophage cells were harvested from adherent culture (in a culture flask, growing at 37 °C in 5% CO_2 ambient). The cells were then incubated with 5000 NPs/cell for 1 hr. Following incubation, the cells were purified once by centrifugation (130g, for 5 minutes) and then we selectively purified only the labelled cells via flow cytometry (BD Aria) to remove any nanoparticle or cell debris. The yield of labelled macrophages via flow-sorting was 30% of total cells. These cells were then fixed in 4% paraformaldehyde for 15 minutes, purified by centrifugation and counted again. We then made spots of 100, 1000 and 10,000 cells on a cover-slip bottom dish. Finally, the cells were imaged on DOLPHIN. We used the HDI mode of imaging,

with excitation at 980 nm with a power density of ~ 100 mW/cm^2, and a long-pass emission filter of 1100 nm.

Letters of "MIT" made by the rare-earth doped nanoparticles:

An "MIT"-shaped feature was generated by applying a coating of up-conversion nanoparticles (UCNPs) on a quartz glass slide. The three letters were coated in three different UCNPs, corresponding to 3 unique wavelengths of emission. The typical dopant concentrations are $NaYF_4$:Yb:X = 78:20:2 atomic %, with the dopant element X = Er for "M", Pr for "I" and Ho for "T", with peak emission at 1,575 nm, 1,375 nm and 1,175 nm, respectively.

Tissues used in this study:

The breast-mimic polymer phantom used in this study is the BioMimic optical phantom provided by INO (reference wavelength 780 nm; absorption coefficient, μ_a, 0.03 cm^{-1}; and reduced scattering coefficient, μ_s', 9 cm^{-1}). The 2 mm and 4 mm thick phantoms have an area of 6 cm × 6 cm, while multiple phantoms of 1 cm and 2 cm thick with an area of 9 cm × 9 cm are used in a stack to achieve a thickness of up to 8 cm.

The bone, fat, skin, and brain tissues are obtained from anatomical parts of a cow from an abattoir (Research 87 INC). The muscular tissue, thin- and thick-cut chicken breast, is obtained from a grocery store. Different thicknesses of fat, brain and muscular tissues are achieved by cutting the tissues with a kitchen knife. Different thicknesses of skin are achieved by stacking layers. The bones are used as received with minor cutting on the edges to obtain a relatively planar geometry.

Mouse handling and injection:

All *in vivo* experiments were performed in compliance with the Institutional Animal Care and Use Committee protocols. Animal experiment procedures were pre-approved (Protocol #1215-112-18) by the Division of Comparative Medicine (DCM) and the Committee on Animal Care (CAC), Massachusetts Institute of Technology, and in compliance with the Principles of Laboratory Animal Care of the National Institutes of Health (NIH), United States of America.

NCr nude mice (4-6 weeks old) are purchased from Taconic and an AIN-76A purified diet is from TestDiet. Mice are kept on the AIN-76A diet for at least a week before *in vivo* imaging experiments to reduce levels of body phosphorescent alfalfa. For tracking fluorescence probes in the

gastrointestinal tract as shown in **Figure 7m**, the mice receive a bolus (a single 100 μm-sized above-mentioned Er-NP/PLA complex is suspended in 20 μl water) through oral administration.

Heartbeat and breathing during live animal imaging:

We did not perform further correction due to heartbeat and breathing. As indicated in the main text, to calculate the probe depths, the point cloud of the top surface of the animal is collected by a 3D scanner, which takes one-half to one minute (depending on the grid size). We consider both the collected top surface profile and the depth calculated from the actual imaging to be affected by the heartbeat and breathing, which leads to a certain amount of inaccuracy in the final results. As such, we did not claim the z-resolution of the system. However, we believe that these heartbeat and breathing factors could be solved using similar methods as in MRI and CT measurements.

Tissue and animal penetration studies, using DOLPHIN:

For the animal depth penetration studies as shown in **Figures 7a-7l**, the mice and rats are euthanized prior to whole-body imaging, and the hair of rat is removed by applying Nair to prevent scattering. The fluorescence probes are placed underneath the animal or the tissue (as shown in **Figure S5a**), while the laser excites the probes directly and the fluorescence signal is detected through the animal or the tissue.

Tissue penetration studies, using conventional NIR-II imaging system:

The tissue penetration studies using a conventional NIR-II imaging system is performed similarly with fluorescence probes placed underneath the tissues, and the fluorescence signal is detected through the tissues[34]. For the results shown in **Figure 6b**, "conventional trans-illumination" is achieved by laser illumination directly to the fluorescence probes; "conventional epi-fluorescence" is achieved by laser illumination from the same direction of the detector, and the laser needs to pass through the tissues before exciting the probes.

Optical properties of biological tissues:

The effective extinction coefficient (μ_{eff}) depends on the absorption coefficient (μ_a) and reduced scattering coefficient (μ_s'), $\mu_{\mathrm{eff}} = \sqrt{3 \times \mu_a \times (\mu_a + \mu_s')}$. μ_a and μ_s' for different types of tissues, as well as the composition of these tissues are adapted from several reviews[5–10].

Supplementary Figures:

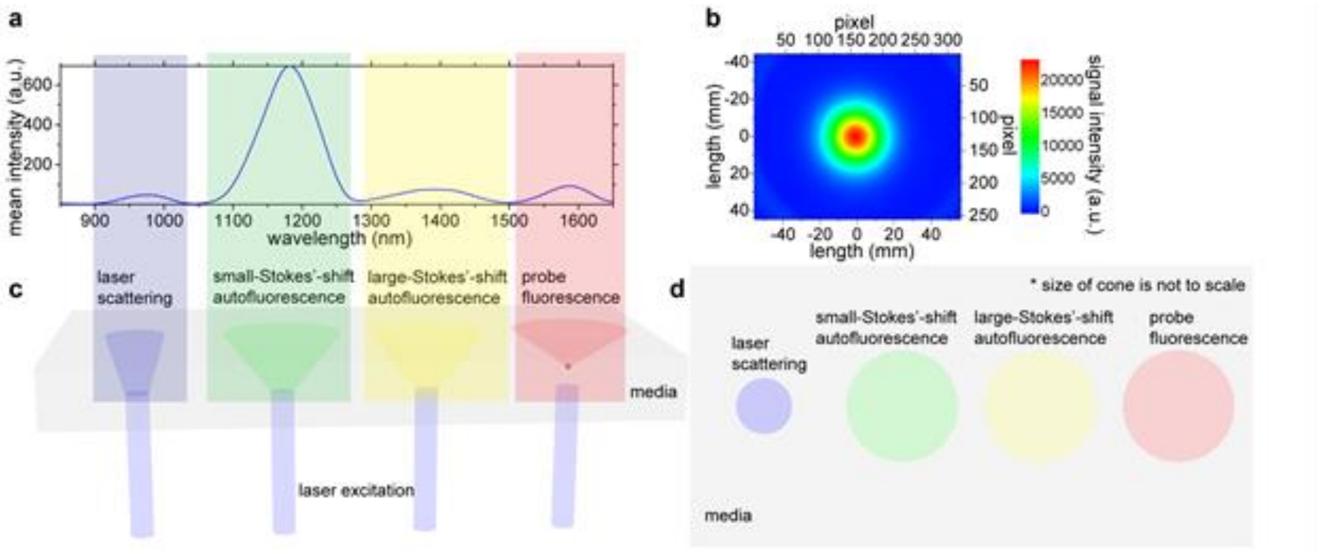

Figure S1. Challenges of fluorescence optical imaging.
(a) A representative overall emission spectrum detected in NIR region, including multiple spectral peaks, which can be attributed to various photo-physical origins; (**b)** a representative 2D emission profile (**b**) detected on the photon-exiting plane; (**c**, **d)** side (**c**) and top (**d**) views of schematic of fluorescence imaging in transilluminating configuration, showing laser incident and scattering (blue cylinders and cone), small- (green cone) and large- (yellow cone) Stokes' shift autofluorescence, and probe fluorescence (red cone).

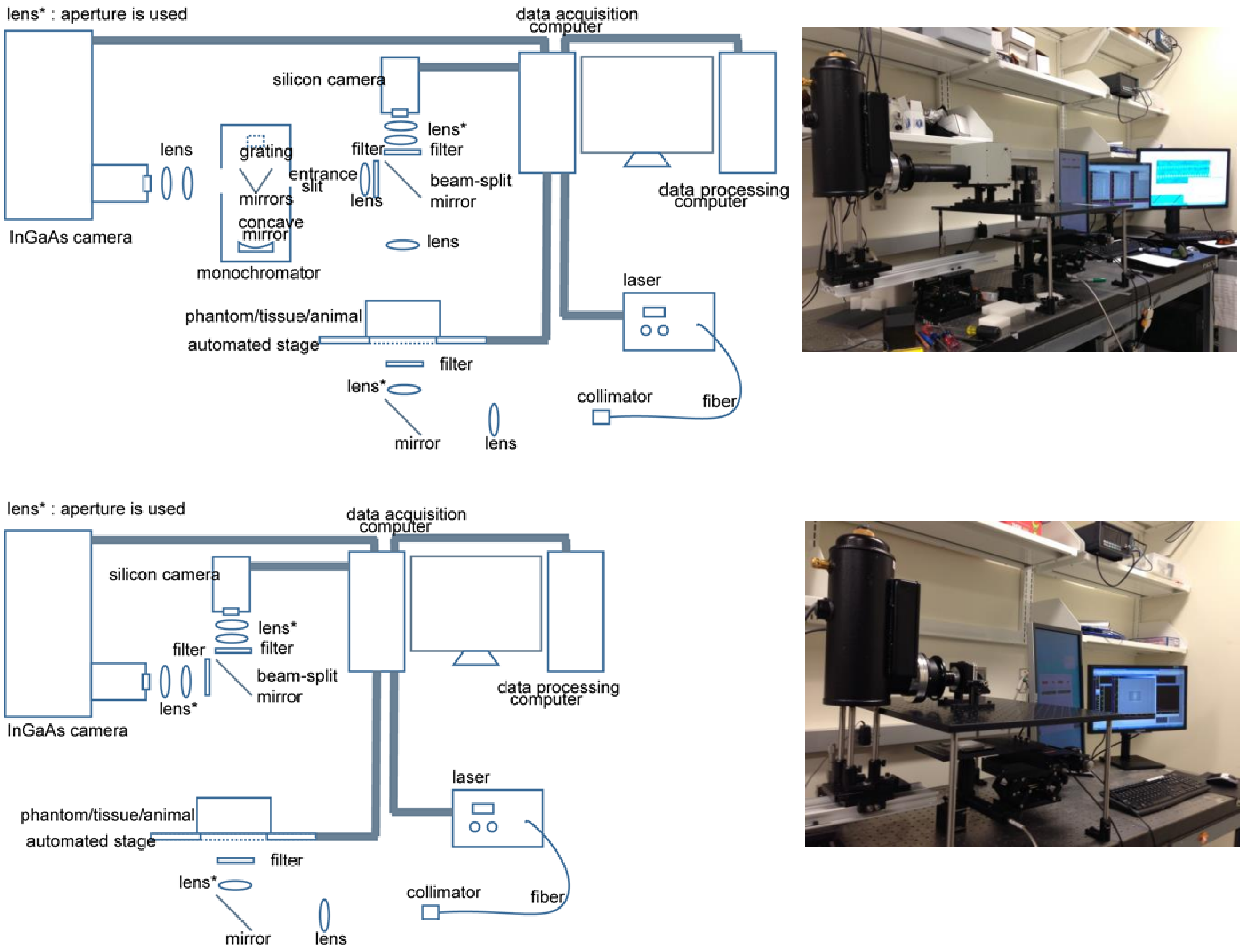

Figure S2. Schematics of HSI and HDI.
(a) The schematic shows DOLPHIN in trans-illumination configuration of HSI mode. The major components include a laser for excitation, an automated *XY* stage, a monochromator for NIR wavelengths, and a liquid nitrogen-cooled InGaAs camera. Hardware control and data acquisition software is written in LabView, and real-time data processing software as well as post processing software are written in MATLAB. Shown to the right is the digital color photograph of the HSI configuration. **(b)** The schematic shows DOLPHIN in trans-illumination configuration of HDI mode. The major difference in HDI mode from HSI mode, is that the NIR fluorescence signal is directly collected by the InGaAs camera without beam-splitting. Shown to the right is the digital color photograph of the HDI configuration.

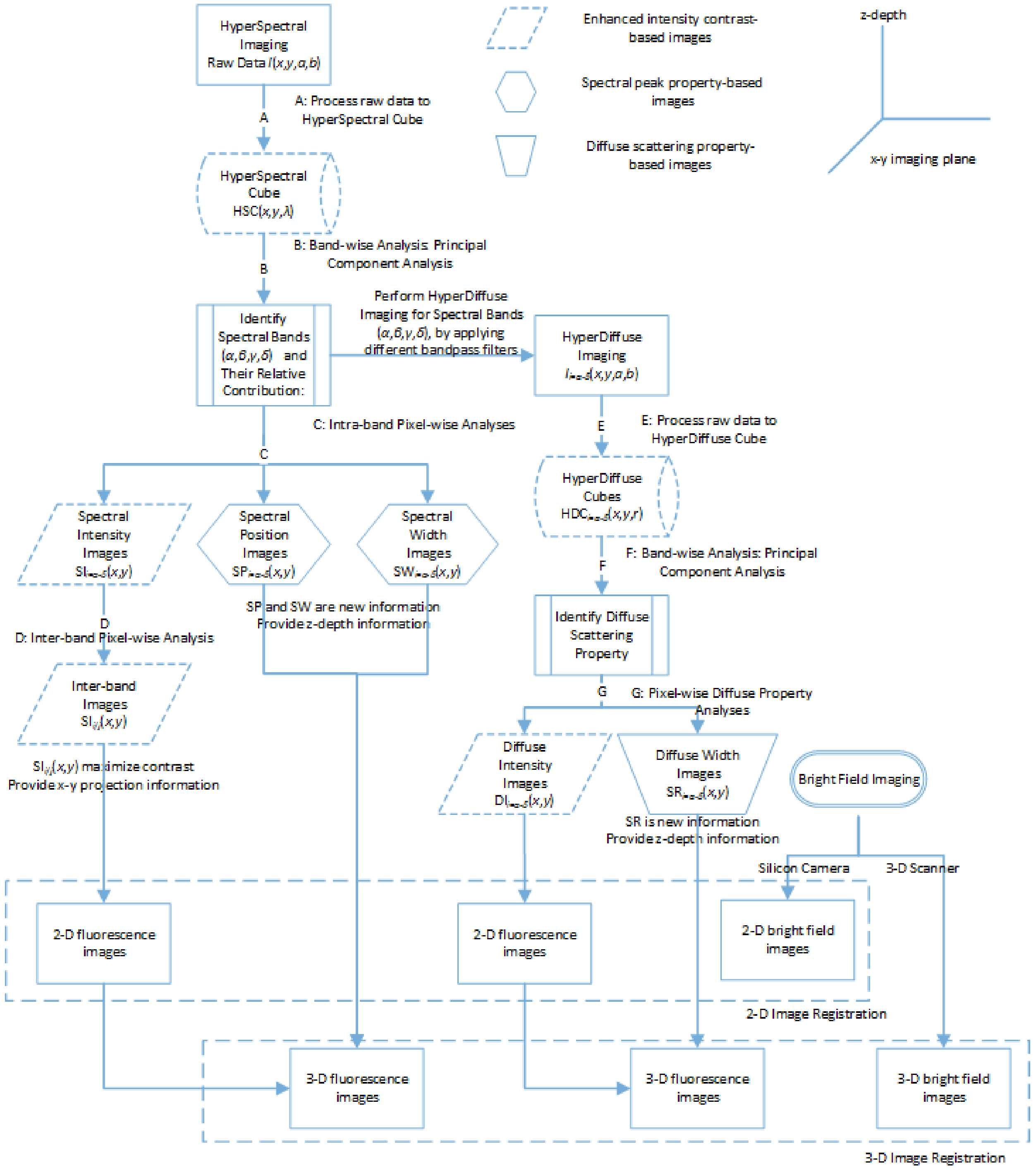

Figure S3. Detailed flowchart of bioimaging with DOLPHIN.
This flowchart describes the work flow of data processing for DOLPHIN. See Figure 2 in the Main text for a simplified version, and also the sub-section "Data Acquisition and Analysis Procedure" under the Results section on pages 6-7 of the Main Text. Please see pages S4-S6 of the Supplementary Methods for a detailed step-by-step description.

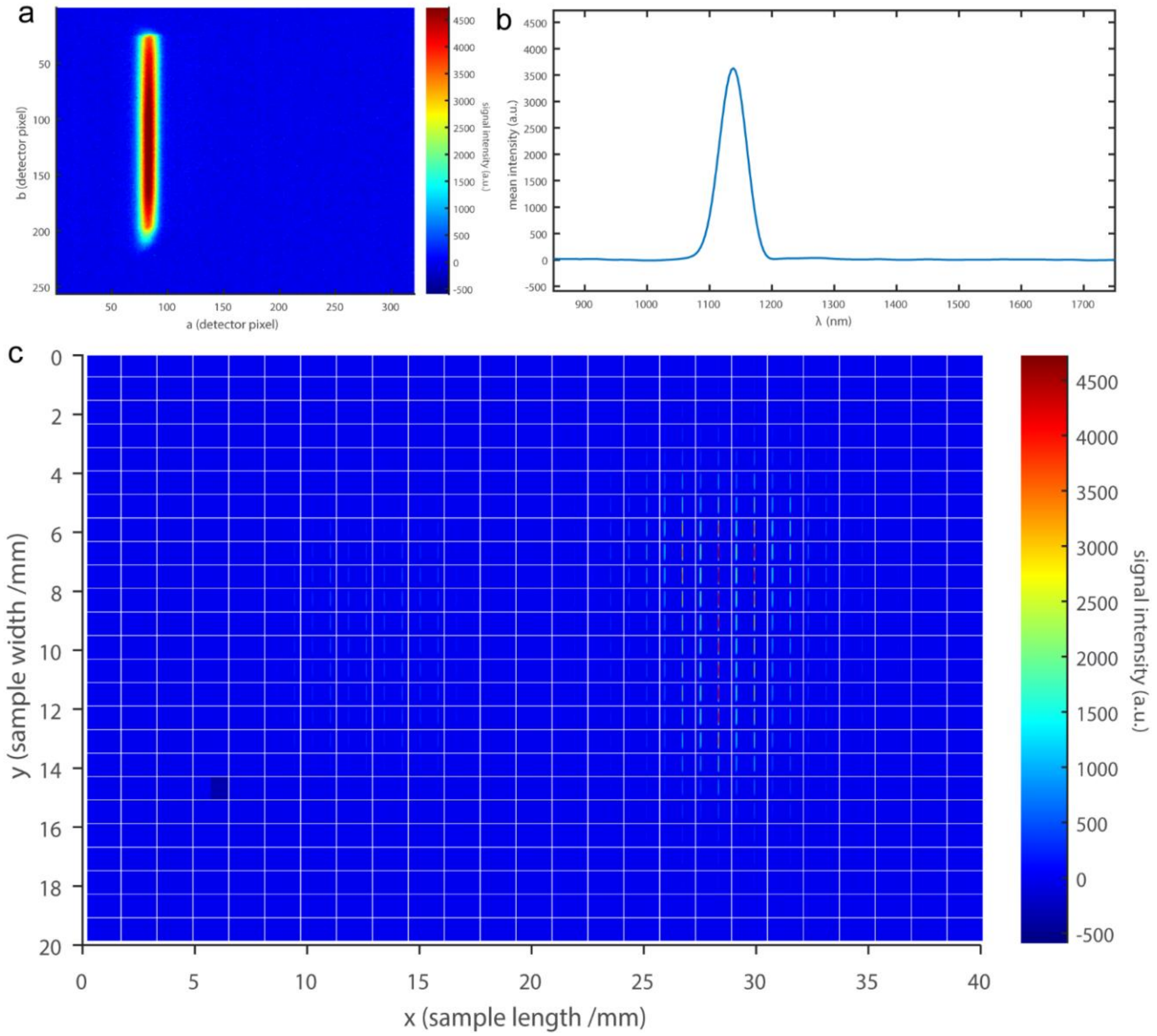

Figure S4. Raw data collected by HSI.

(**a, b)** At each scanning position (x, y), the collected raw data $I(a, b)$ (**a**) and the derived spectrum $I(\lambda)$ (**b**); **c**, the collected raw data at all scanning positions $I(x, y, a, b)$, at each position (x, y) the captured image $I(a, b)$ by the InGaAs camera is plotted, however to visualize the 200 × 100 of 320 × 256 images, a binning factor of 4 on all four dimensions is used to reduce the image size. It is noted that, each small square in (**c)** can be enlarged to the large captured image as shown in (**a)**. In fact, a general collection of raw data **c** contains 200 × 100 squares of (**a)**.

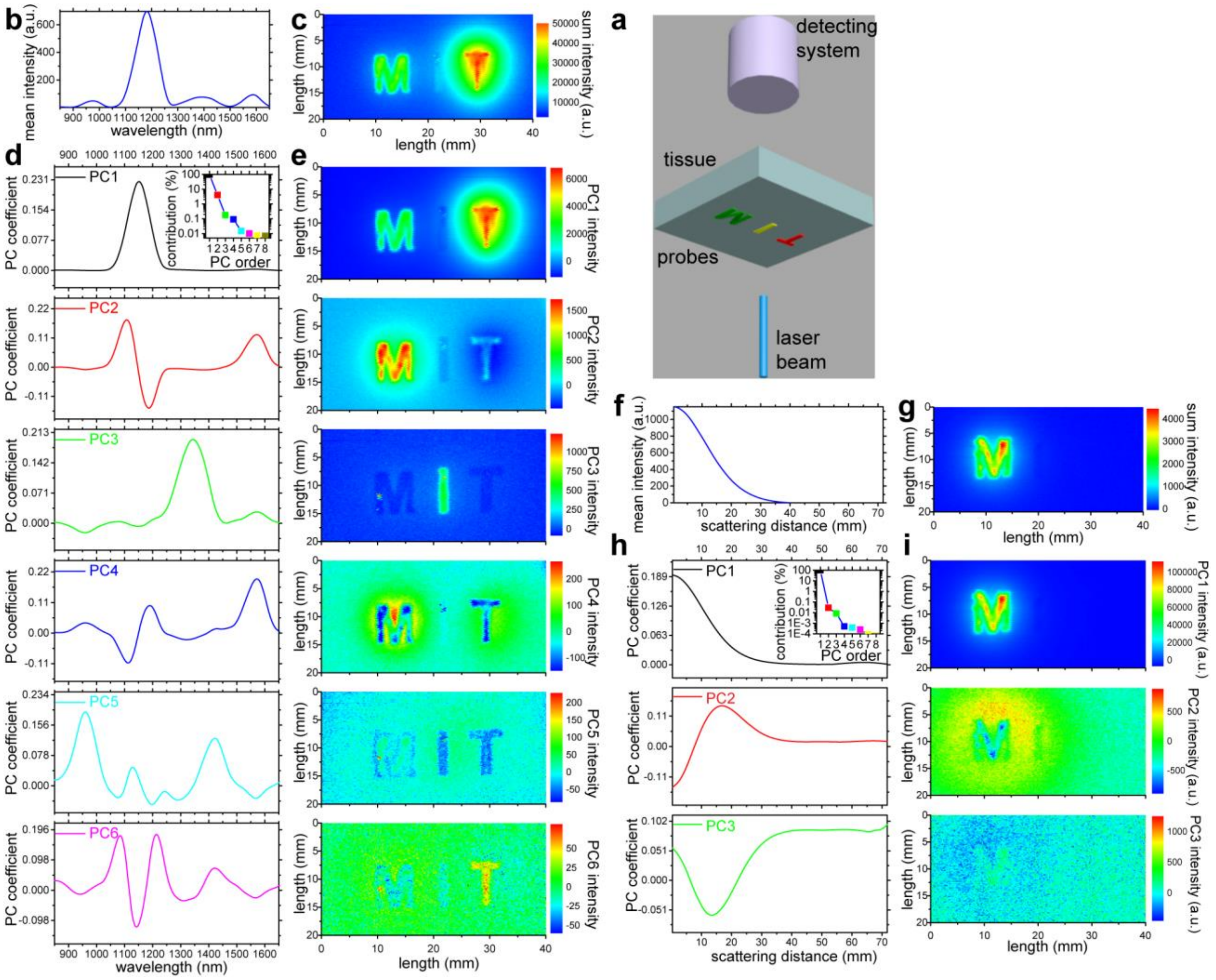

Figure S5. Principal Component Analysis (PCA) of HSC and HDC for tissue penetration.
(a) Schematic of the experiment setup for tissue penetration. The letters of "M", "I", and "T" are made of $NaYF_4$:Yb:Er, $NaYF_4$:Yb:Pr, and $NaYF_4$:Yb:Ho nanoparticles respectively. Various thicknesses of different types of tissues are placed on top of these probes. The laser beam is incident normally on the imaging plane from the bottom to top, and the detector captures the transmitted fluorescence signal on the photon-exiting plane. (**b**, **f)** The overall spectral and scattering features by combining information from the entire spatial domain for HSC (**b**) and HDC (**f**). (**c**, **g)** The overall spatial features by combining information from the entire spectral and scattering domains for HSC (**c**) and HDC (**g**). (**d**, **e**, **h**, **i)** Principal Components, or PCs (**e**, **i**) and the corresponding PC coefficients in frequency (**d**) or scattering (**h**) domain for HSC (**d**, **e**) and HDC (**h**, **i**). Insets of (**d**, **h)**: the relative contributions from PCs of HSC (**d**) and HDC (**h**).

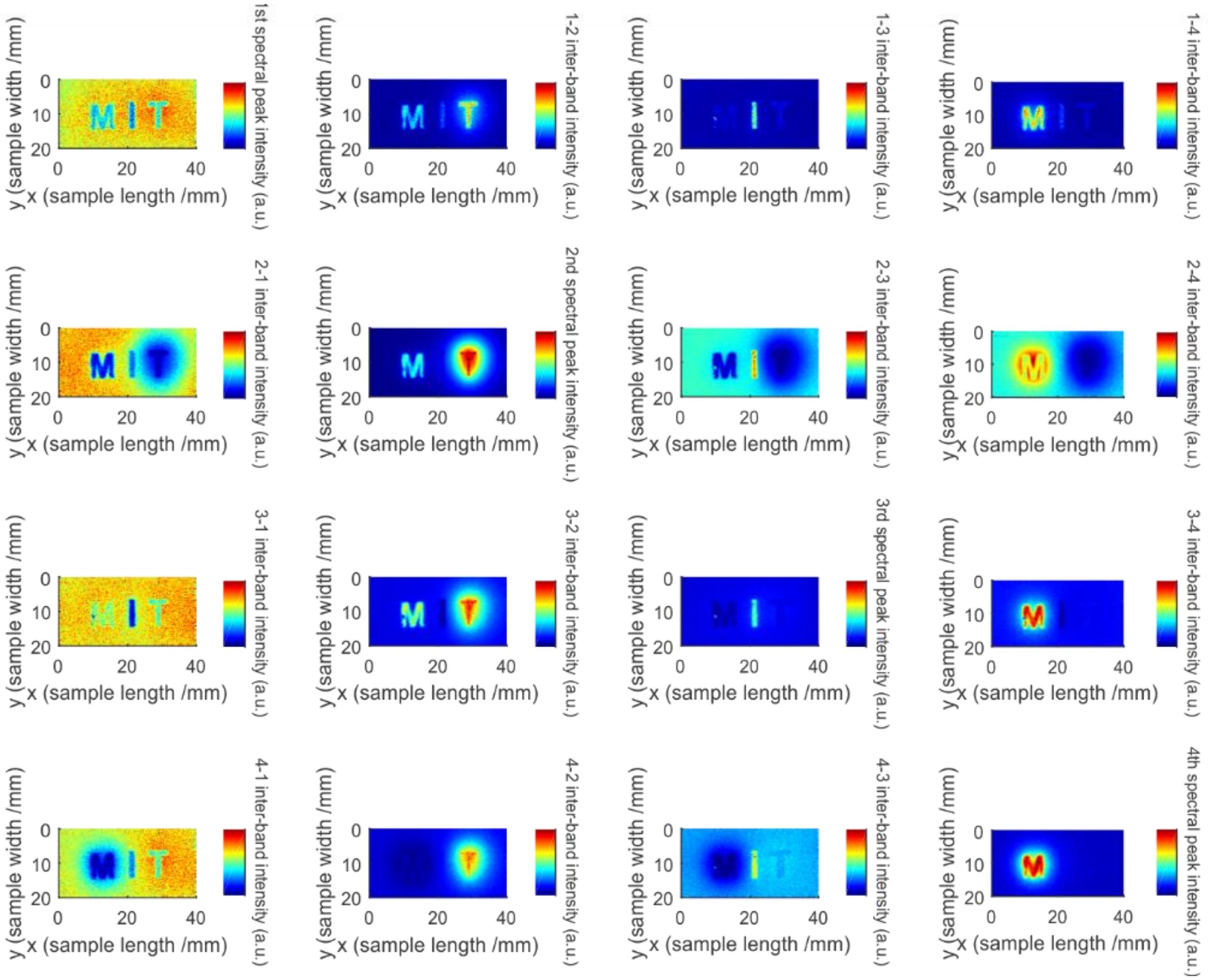

Figure S6. Inter-band analysis of HSI.
The plots represent the inter-band pixel-wise analyses performed on the HSC data; $SI_{i/j}(x, y)$ for $i, j = \alpha, \beta, \gamma$, and δ. When $i = j$, the diagonal elements correspond to the four original SIs for the four spectral bands. The non-diagonal elements provide new information, and this inter-band analysis is used to maximize the image contrast based on the knowledge of origin of contrast of each spectral region. For example, the non-diagonal element, representing $SI_{i/j}$ offers a much sharper resolution, and consequently a better visualization, of the features of interest, in this case the "MIT" feature, compared to the blurring observed in some of the diagonal components. Refer to page S5 for definition of $SI_{i/j}$.

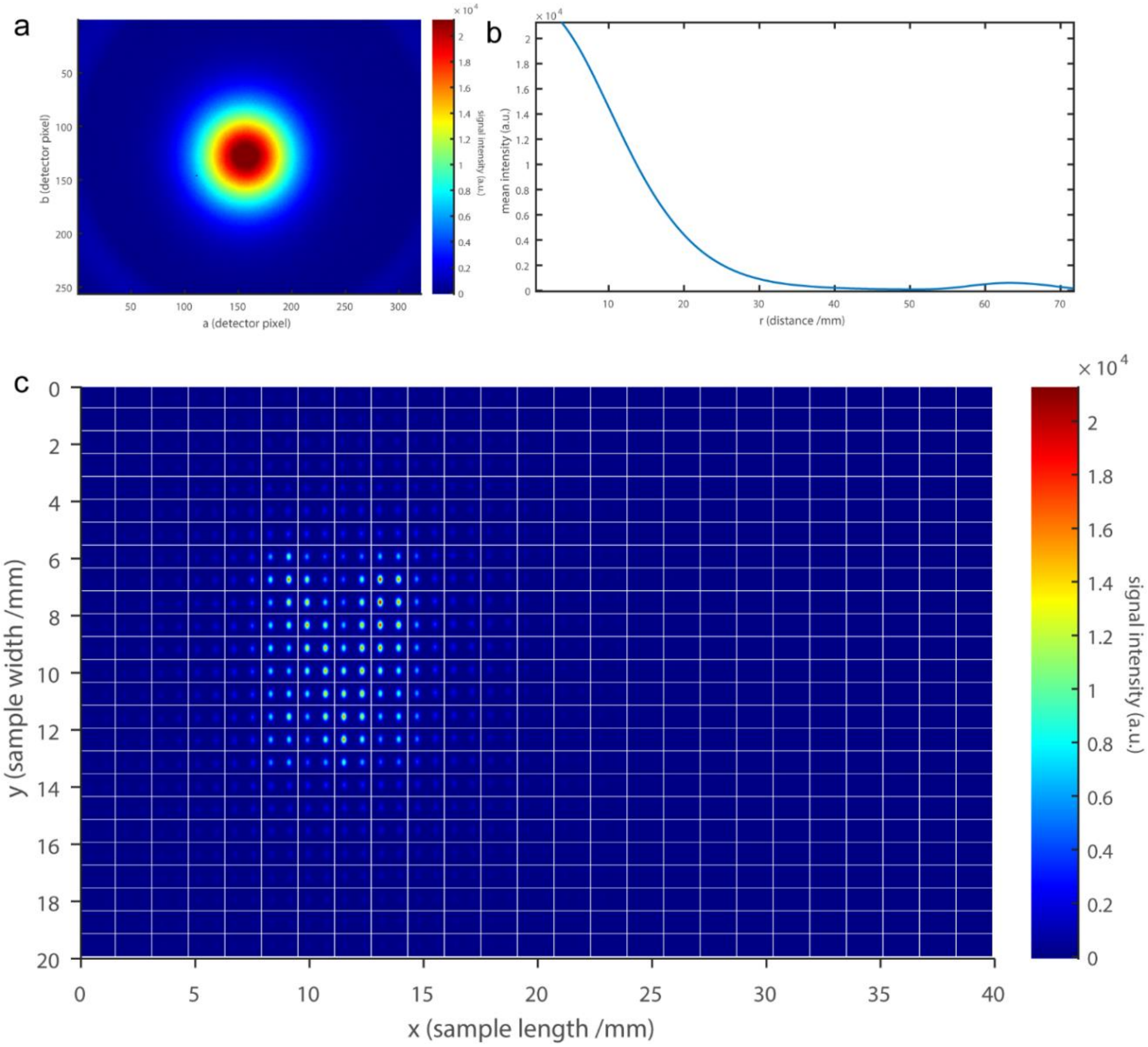

Figure S7. Raw data collected by HDI.
(**a, b)** At each scanning position (*x*, *y*), the collected raw data *I*(*a*, *b*) (**a**) and the derived relation of intensity as a function of scattering distance *I*(*r*) (**b**). (**c)** The collected raw data at all scanning positions *I*(*x*, *y*, *a*, *b*), at each position (*x*, *y*) the captured image *I*(*a*, *b*) by the InGaAs camera is plotted, however to visualize the 200 × 100 of 320 × 256 images, a binning factor of 4 on all four dimensions is used to reduce the image size.

In HDI mode, a similar procedure for data analysis is performed. The key difference here is that based upon the analysis of the HSI data, a suitable optical filter is selected to perform HDI imaging. For the three "M," "I," and "T" features listed above, the combination filters used are (1,400 nm long-pass × 2 + 1,575 nm ± 25 nm band-pass × 2), (1,300 nm long-pass × 2 + 1,375 nm ± 25 nm band-pass × 2) and (1,100 nm long-pass × 2 + 1,150 nm ± 25 nm band-pass × 2) respectively. An example is shown here, for HDI imaging of the "M" letter only.

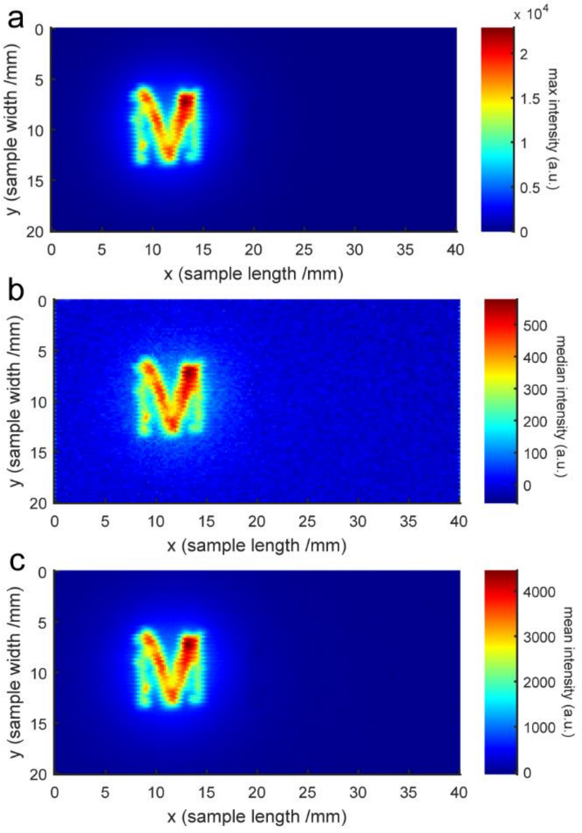

Figure S8. Statistical operations on HDC to extract intensity information while excluding the scattering effect.
Statistical operations of max value **(a)**, median value **(b)**, and mean value **(c)** for HDC (the statistical operations are performed on the scattering dimension) are presented. It is noted that the mean value operation **(c)** shows the same result as **Figure S5g**, which is the summation operation. Each of these operations shows slightly different contrast. Depending on the SNR of the actual data, one of them will have the best contrast.

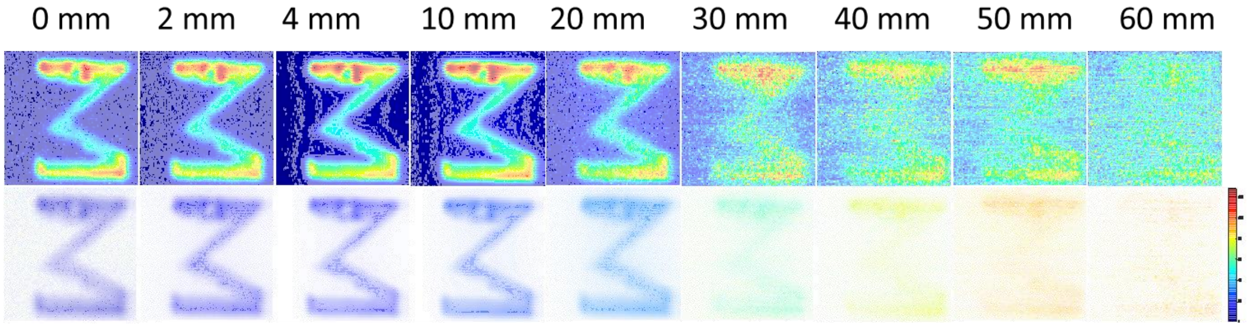

Figure S9. Effect of dispersion on penetration depth from HDC data.
The images were taken by placing the "M" letter, formed by the Er-NP, under varying depths of the breast-mimic tissue phantom, from 0 to 60 mm. The top row shows the processed intensity image, while the bottom row shows the processed dispersion effect image, which shows information of the depth.

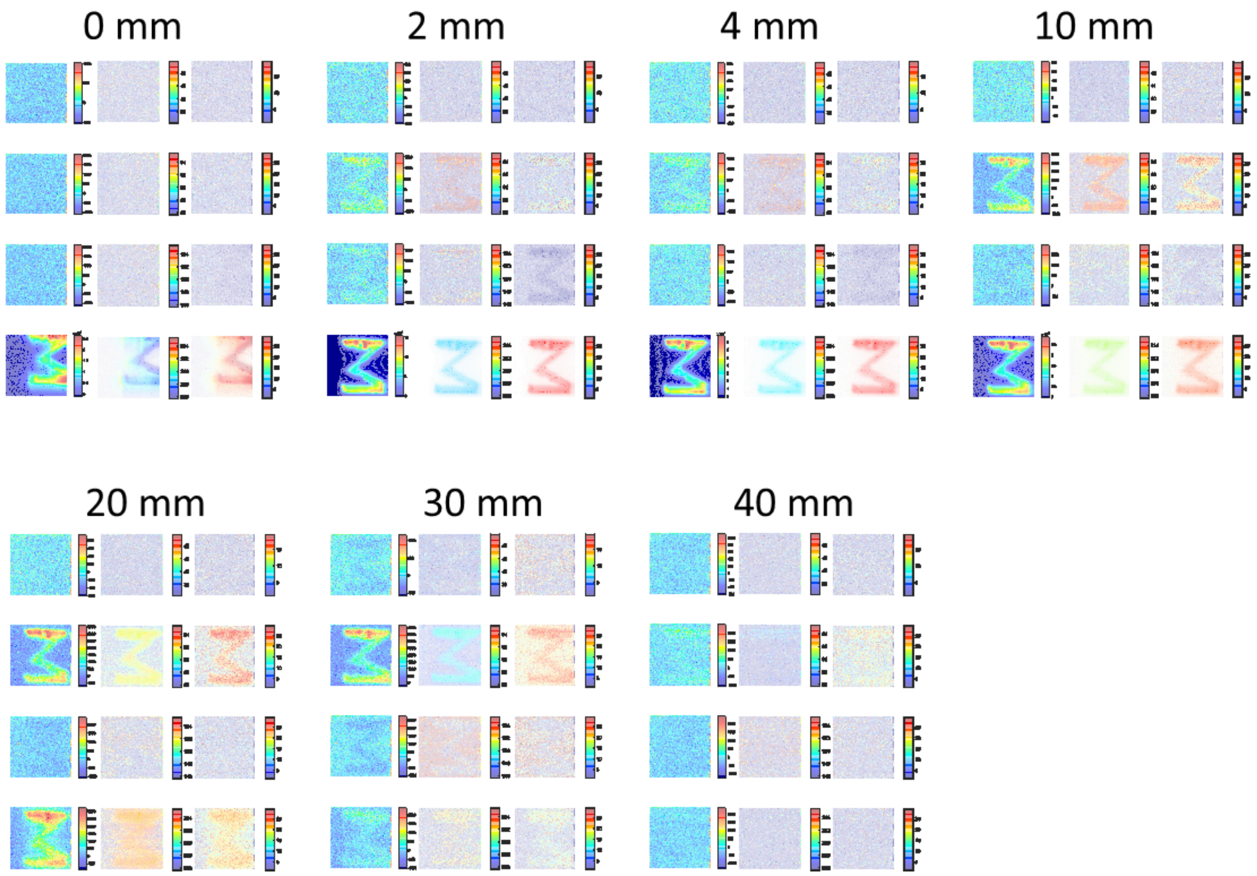

Figure S10. Analysis of spatial scanning and hyperspectral data (HSC) shows information of depth.
In each image, the first column represents SI, the second column represents SP and the third column represents SW images. The four rows represent the 4 bands, α, β, γ, and δ. Shown are the results of data processing for the letter "M" (formed by the Er-NP) placed under varying depths of tissue phantom, 0 to 40 mm. It is interesting to note that for depths of 0 - 20 mm, the δ-band is more prominent, while for depths > 30 mm, the β-band dominates.

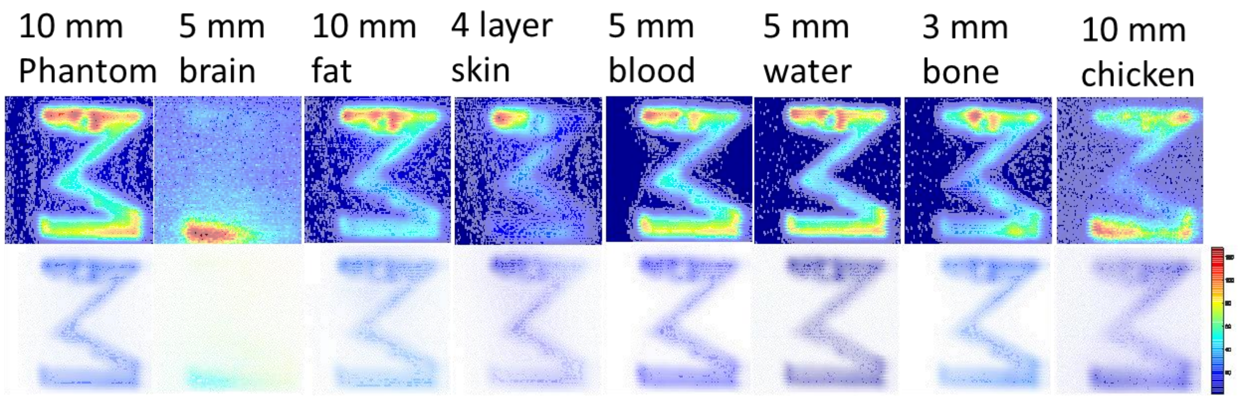

Figure S11. Effect of type of tissue on the dispersion effect through identical penetration depth.
The images were taken by placing the "M" letter, formed by the Er-NP, under nearly identical depths of the various types of tissues studied, including breast-mimic tissue phantom, chicken breast, brain, fat, skin, bone, blood and water. The top row shows the processed intensity image, while the bottom row shows the processed dispersion effect image, which shows information of the depth.

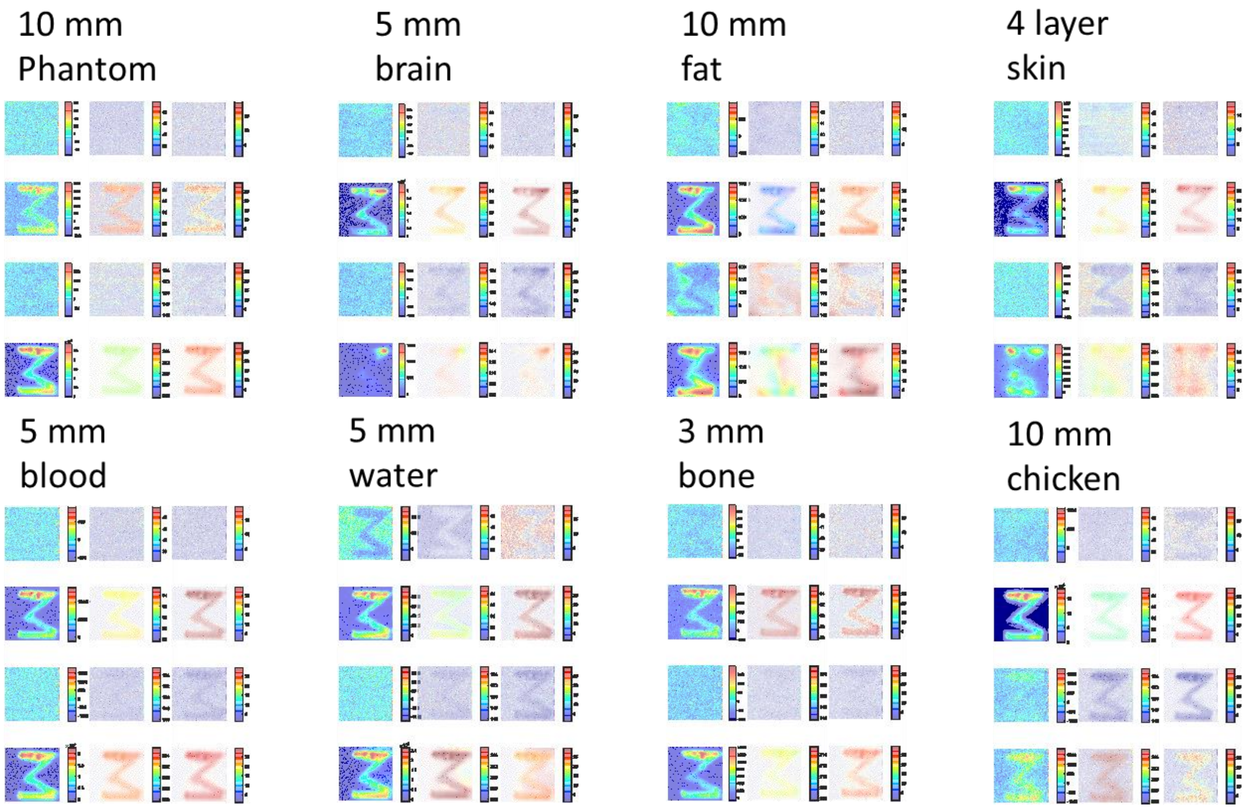

Figure S12. Effect of tissue environment on the in-band and inter-band characteristics from HSC data. In each image, the first column represents SI, the second column represents SP and the third column represents SW images. The four rows represent the 4 bands, α, β, γ, and δ. Shown are the results of data processing for the letter "M" (formed by the Er-NP) placed under nearly similar depths (5 - 10 mm) of tissue phantom, brain, fat, skin, blood, water, bone and chicken breast. It is interesting to note that for different types of tissue, a specific image processing technique at a specific band dominates (in terms of providing the best visual contrast). For example, the SI image of the β-band provides the best contrast for brain tissue, while SI or SW of the δ-band may offer the best visual contrast for fat tissue or tissue phantom.

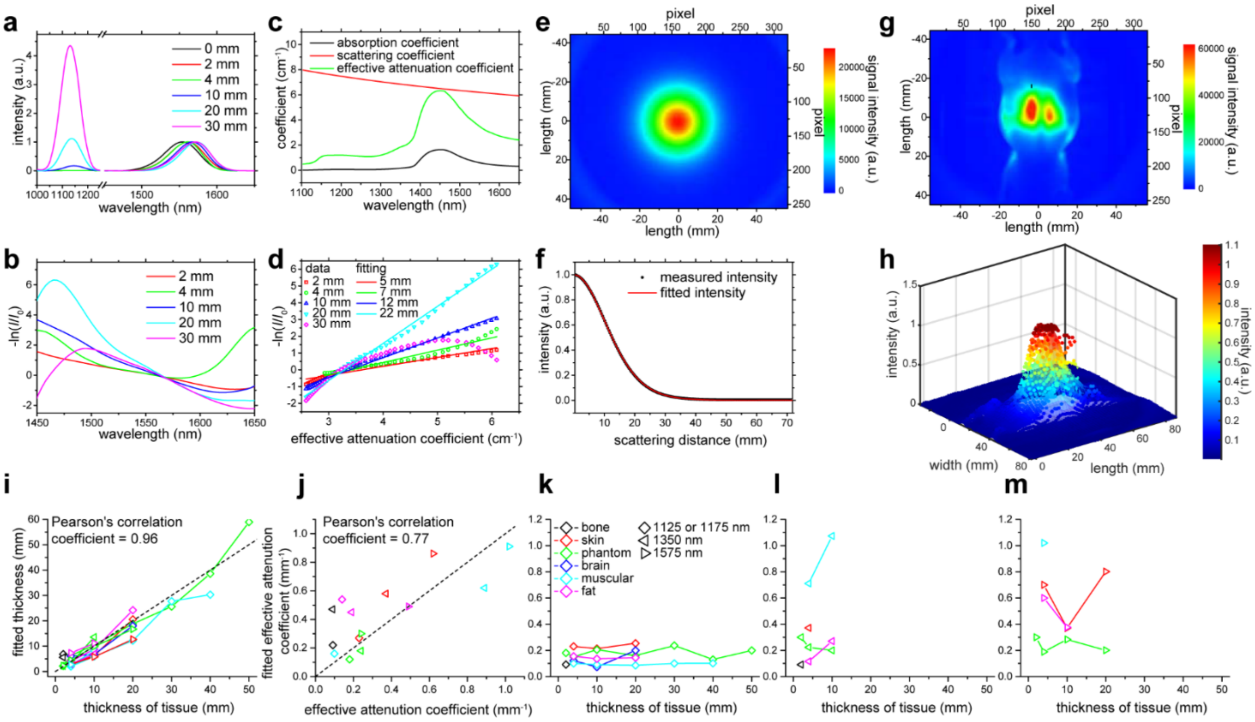

Figure S13. Deriving depth of signal and effective attenuation coefficient of tissues from fitting the results of tissue or animal penetration by DOLPHIN.

(**a**, **b)** The emission spectra normalized at Er-1575 peak (**a**) and -ln(I/I_0)) (**b**), where I and I_0 are the transmitted emission intensity through tissue and the intrinsic emission intensity) of Er-NP measured from HSI, penetrating through 0-30 mm of breast-mimic phantom. (**c)** The estimated absorption coefficient, scattering coefficient, and effective attenuation coefficient of breast tissue. (**d)** Fitting of tissue depth using Beer's law and data presented in (**b**, **c)**. (**e**) Measured by HDI, the 2D scattering profile detected from photon-exiting plane for a 2 cm-thick breast-mimic phantom. The scattering profile shows cylindrical symmetry. (**f)** The corresponding 1D scattering profile as a function of scattering distance (data points in black), and the fitted results using depth and effective attenuation coefficient as fitting parameters (red line), which is in excellent agreement with the measured data. (**g)** A representative 2D scattering profile measured by HDI for a whole mouse. The scattering profile shows no cylindrical symmetry. Similar to the tissue penetration experiment, the fluorescence probe of Er-NP is placed directly underneath the mouse at the location with maximum height. (**h)** The measured (shown as 3D scattered points) and fitted results (shown as solid surface profile) for the mouse imaging using the generalized fitting equation with depth and effective attenuation coefficient as the fitting parameters. (**i**, **j)** The fitted thicknesses (**i**) and effective attenuation coefficients (**j**) compared to the actual thicknesses of the tissues and the estimated effective attenuation coefficients. The black dashed lines in (**i, j)** represent equivalency between fitted and actual values. (**k-m)** The fitted effective attenuation coefficients for various tissues and thicknesses at wavelengths of 1125 nm or 1175 nm (**k**), 1350 nm (**l**), 1575 nm (**m**). The plots in (**i-m)** share the same legends shown in (**k**).

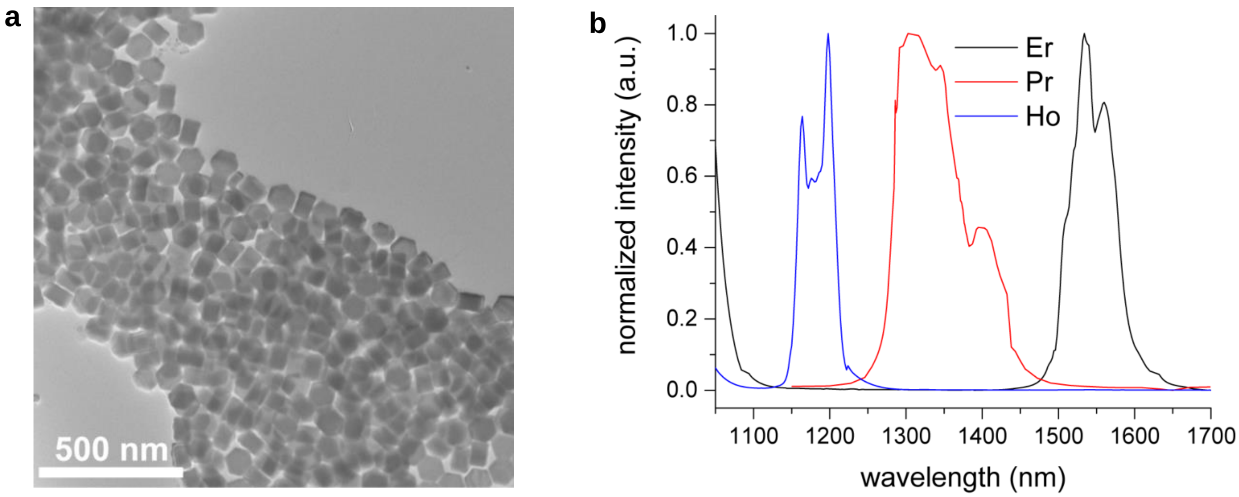

Figure S14. Transmission electron micrograph (TEM) and down-conversion fluorescence spectra of the rare-earth nanoparticles.

(a) The nanoparticles are approximately 60 nm in the transmission electron micrograph, which ensures efficient fluorescence and are sufficiently small for biomedical applications. The NPs are hexagonal plates in shape (appear hexagonal in top view, and flat in side view). (**b)** The spectral peaks of 1,575 nm from Er-NP, 1,175 nm from Ho-NP, and 1,350 nm from Pr-NP are clearly observed. However, the spectral peak of 1,125 nm from Er-NP is observed as part of the descending slope overlapping with the scattered light from excitation. The observed 1,125 nm from Er-NP by DOLPHIN is the combined outcome of the emission of the particle close to excitation wavelength (980 nm laser) and the 1,100 nm longpass cut off filters (to avoid damage to the camera from excitation light).

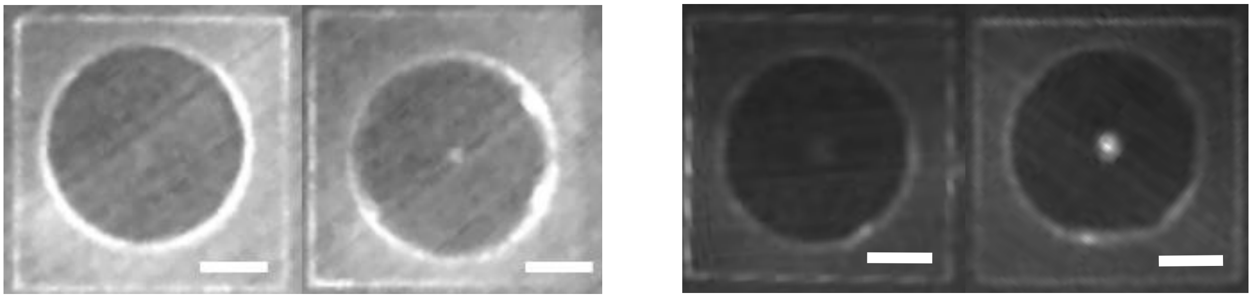

Figure S15. Sample image of cells labeled with Er-doped NPs, using DOLPHIN.

Macrophages (RAW 264.7) were incubated with 5000 NPs per cell for 1 hr. at 37 °C, purified using flow cytometry, and imaged after fixation. **(Left panel)** 100 macrophage cells (*left*, control – cells only, no NPs, *right*, labeled with NPs), **(Right panel)** 1,000 macrophage cells (*left*, control – cells only, no NPs, *right*, labeled with NPs). Scale bar is 4.5 mm for all images.

Supplementary movies:

Movie S1. Process of data collection of DOLPHIN.

In particular, this movie shows the process of data collection during HSI. The arrangement of the three sections is identical to **Figure S4**. As the laser scans through the sample area, at each scanning position (x, y), the collected raw data $I(a, b)$ and the derived spectrum $I(\lambda)$ are shown. The collected raw data at all scanning positions $I(x, y, a, b)$, at each position (x, y) the captured image $I(a, b)$ by the InGaAs camera is plotted, however to visualize the 200 × 100 of 320 × 256 images, a binning factor of 4 on all four dimensions is used to reduce the image size. For HDI, the process of data collection is similar, as raw data $I(a, b)$ at each position (x, y) converts to $I(r)$.

Movie S2. 3D visualization of HSC.

As the HSC, $I(x, y, \lambda)$, is achieved by data analysis, $I(x, y)$ at all λ are plotted in the order of increasing wavelength. On the left is the visualization of HSC slice by slice in 3D, and on the right is the enlarged view of each slice. This movie can be considered as combining the information from **Figures S5** and **S6**.

Movie S3. Fluorescence 3D reconstruction of 100 μm sized Er-NP visualized under a whole mouse.

In this movie, the camera moves from elevation angle of 90° to 0° to show the 3D position of the fluorescent particle relative to the anatomy of the mouse.

Supplementary Code:

All the MATLAB files associated with the data processing used in this work are made available publicly, in this GitHub repository: https://github.com/belcherlab/DOLPHIN (under the MIT License). Please read the README.txt file in this repository for instructions and file descriptions.

A small test dataset is made available on the Data Dryad archive.

Supplementary References

1. Auzel, F. Upconversion and Anti-Stokes Processes with f and d Ions in Solids. *Chem. Rev.* **104**, 139–174 (2004).
2. van der Meer, F. D. *et al.* Multi- and hyperspectral geologic remote sensing: A review. *Int. J. Appl. Earth Obs. Geoinformation* **14**, 112–128 (2012).
3. Lu, G. & Fei, B. Medical hyperspectral imaging: a review. *J. Biomed. Opt.* **19**, 010901–010901 (2014).
4. Durduran, T., Choe, R., Baker, W. B. & Yodh, A. G. Diffuse optics for tissue monitoring and tomography. *Rep. Prog. Phys.* **73**, 076701 (2010).
5. Bashkatov, A. N., Genina, E. A., Kochubey, V. I. & Tuchin, V. V. Optical properties of human skin, subcutaneous and mucous tissues in the wavelength range from 400 to 2000 nm. *J. Phys. Appl. Phys.* **38**, 2543–2555 (2005).
6. Bashkatov, A. N., Genina, E. A., Kochubey, V. I. & Tuchin, V. V. Optical properties of human cranial bone in the spectral range from 800 to 2000 nm. in *Proc. SPIE 6163, Saratov Fall Meeting 2005: Optical Technologies in Biophysics and Medicine VII* (ed. Tuchin, V. V.) 616310--1–616310--11 (SPIE, 2006). doi:10.1117/12.697305
7. Cheong, W.-F., Prahl, S. A. & Welch, A. J. A review of the optical properties of biological tissues. *IEEE J. Quantum Electron.* **26**, 2166–2185 (1990).
8. Jacques, S. L. Optical properties of biological tissues: a review. *Phys. Med. Biol.* **58**, R37 (2013).
9. Roggan, A., Friebel, M., Do Rschel, K., Hahn, A. & Mu Ller, G. Optical Properties of Circulating Human Blood in the Wavelength Range 400-2500 nm. *J. Biomed. Opt.* **4**, 36–46 (1999).

10. Sandell, J. L. & Zhu, T. C. A review of *in-vivo* optical properties of human tissues and its impact on PDT. *J. Biophotonics* **4**, 773–787 (2011).

11. Mackenzie, L. E. *et al.* The theoretical molecular weight of $NaYF_4$:RE upconversion nanoparticles. *Sci. Rep.* **8**, 1106 (2018).

12. Strohhöfer, C. & Polman, A. Absorption and emission spectroscopy in Er^{3+}–Yb^{3+} doped aluminum oxide waveguides. *Opt. Mater.* **21**, 705–712 (2003).

13. Nadort, A. *et al.* Quantitative Imaging of Single Upconversion Nanoparticles in Biological Tissue. *PLOS ONE* **8**, e63292 (2013).

14. Wu, H. *et al. In vivo* lipidomics using single-cell Raman spectroscopy. *Proc. Natl. Acad. Sci.* **108**, 3809–3814 (2011).

15. del Rosal, B., Villa, I., Jaque, D. & Sanz-Rodríguez, F. *In vivo* autofluorescence in the biological windows: the role of pigmentation. *J. Biophotonics* **9**, 1059–1067 (2016).

16. Zavattini, G. *et al.* A hyperspectral fluorescence system for 3D *in vivo* optical imaging. *Phys. Med. Biol.* **51**, 2029 (2006).

17. Randeberg, L. L., Baarstad, I., Løke, T., Kaspersen, P. & Svaasand, L. O. Hyperspectral imaging of bruised skin. in *Proc. SPIE 6078, Photonics Therapeutics and Diagnostics II* (eds. Kollias, N. et al.) 60780O-60780O–11 (SPIE, 2006). doi:10.1117/12.646557

18. Usenik, P., Bürmen, M., Fidler, A., Pernuš, F. & Likar, B. Evaluation of cross-polarized near infrared hyperspectral imaging for early detection of dental caries. in *Proc. SPIE 8208, Lasers in Dentistry XVIII* **8208**, 82080G-82080G–7 (SPIE, 2012).

19. Randeberg, L. L. & Hernandez-Palacios, J. Hyperspectral imaging of bruises in the SWIR spectral region. in *Photonic Therapeutics and Diagnostics VIII* **8207**, 82070N (International Society for Optics and Photonics, 2012).

20. Panasyuk, S. V. *et al.* Medical hyperspectral imaging to facilitate residual tumor identification during surgery. *Cancer Biol. Ther.* **6**, 439–446 (2007).

21. Zuzak, K. J. *et al.* Intraoperative bile duct visualization using near-infrared hyperspectral video imaging. *Am. J. Surg.* **195**, 491–497 (2008).

22. Ntziachristos, V. Fluorescence Molecular Imaging. *Annu. Rev. Biomed. Eng.* **8**, 1–33 (2006).

23. Ntziachristos, V., Tung, C.-H., Bremer, C. & Weissleder, R. Fluorescence molecular tomography resolves protease activity *in vivo*. *Nat Med* **8**, 757–761 (2002).

24. Bai, J. & Xu, Z. Fluorescence Molecular Tomography. in *Molecular Imaging - Fundamentals and Applications* 185–216 (Springer-Verlag Berlin Heidelberg, 2013).

25. Milstein, A. B. *et al.* Fluorescence optical diffusion tomography. *Appl. Opt.* **42**, 3081–3094 (2003).

26. Ntziachristos, V. & Weissleder, R. Experimental three-dimensional fluorescence reconstruction of diffuse media by use of a normalized Born approximation. *Opt. Lett.* **26**, 893–895 (2001).

27. Corlu, A. *et al.* Three-dimensional *in vivo* fluorescence diffuse optical tomography of breast cancer in humans. *Opt. Express* **15**, 6696–6716 (2007).

28. Ntziachristos, V., Bremer, C. & Weissleder, R. Fluorescence imaging with near-infrared light: new technological advances that enable *in vivo* molecular imaging. *Eur. Radiol.* **13**, 195–208 (2003).

29. Ye, X. *et al.* Morphologically controlled synthesis of colloidal upconversion nanophosphors and their shape-directed self-assembly. *Proc. Natl. Acad. Sci.* **107**, 22430–22435 (2010).

30. Würth, C., Grabolle, M., Pauli, J., Spieles, M. & Resch-Genger, U. Relative and absolute determination of fluorescence quantum yields of transparent samples. *Nat. Protoc.* **8**, 1535–1550 (2013).

31. Naczynski, D. J. *et al.* Rare-earth-doped biological composites as *in vivo* shortwave infrared reporters. *Nat. Commun.* **4**, 2199 (2013).

32. Wang, M., Abbineni, G., Clevenger, A., Mao, C. & Xu, S. Upconversion nanoparticles: synthesis, surface modification and biological applications. *Nanomedicine Nanotechnol. Biol. Med.* **7**, 710–729 (2011).

33. Smith, A. M., Mancini, M. C. & Nie, S. Bioimaging: Second window for *in vivo* imaging. *Nat. Nanotechnol.* **4**, 710–711 (2009).

34. Yi, H. *et al.* M13 Phage-Functionalized Single-Walled Carbon Nanotubes As Nanoprobes for Second Near-Infrared Window Fluorescence Imaging of Targeted Tumors. *Nano Lett* **12**, 1176–1183 (2012).
